# Twelve thousand recent patellogastropods from a northeastern Pacific latitudinal gradient

**DOI:** 10.1038/sdata.2017.197

**Published:** 2018-01-09

**Authors:** Sara S. Kahanamoku, Pincelli M. Hull, David R. Lindberg, Allison Y. Hsiang, Erica C. Clites, Seth Finnegan

**Affiliations:** 1 Yale University, Department of Geology & Geophysics, New Haven, CT 06511, USA; 2 University of California, Department of Integrative Biology and Museum of Paleontology, Berkeley, CA 94720, USA; 3 Swedish Museum of Natural History, Department of Bioinformatics and Genetics, Stockholm 10405, Sweden; 4 University of California Museum of Paleontology, Berkeley, CA 94720, USA

**Keywords:** Community ecology, Macroecology, Marine biology

## Abstract

Body size distributions can vary widely among communities, with important implications for ecological dynamics, energetics, and evolutionary history. Here we present a dataset of body size and shape for 12,035 extant Patellogastropoda (true limpet) specimens from the collections of the University of California Museum of Paleontology, compiled using a novel high-throughput morphometric imaging method. These specimens were collected over the past 150 years at 355 localities along a latitudinal gradient ranging from Alaska to Baja California, Mexico and are presented here with individual images, 2D outline coordinates, and 2D measurements of body size and shape. This dataset provides a resource for assemblage-scale macroecological questions and documents the size and diversity of recent patellogastropods in the northeastern Pacific.

## Background & Summary

Body size is one of the most important aspects of organismal form and function, and influences a broad array of physiological, ecological, and evolutionary processes^1^. Environmental controls on body size have been studied in many different groups for over a century^2,3^, particularly along latitudinal gradients, which serve as natural experiments in which to study the effects of abiotic change on organismal and assemblage size distributions^4,5^. There is a large amount of variance in body size distributions at all scales, which may be difficult to examine using traditional methods, many of which are time-intensive. Automated methods of measuring body size, developed more recently, have the potential to incorporate individual data from large numbers of specimens. Speeding up morphological measurements helps to facilitate the collection of large datasets that capture the full range of variation within and among communities, which may then be used to elucidate the factors that determine body size distributions across space and time^6^. Here we present a comprehensive database of the size and shape of Patellogastropoda (true limpets) from Baja California, Mexico to Alaska. Patellogastropods are a globally distributed group, comprising ~400 species^7^, that are nearly ubiquitous in intertidal habitats. They thus provide an interesting comparison to fully marine groups such as bivalves that have been the focus of previous analyses of marine body size distributions example refs 3,6. Further, because they may be affected by changes in both atmospheric temperature and the temperature and pH of ocean waters^8–10^, establishing baseline gradients in patellogastropod body size can help to detect and diagnose the causes of future changes in the spatial structure of body size distributions^11,12^.

Breakthroughs in imaging and digitization^13^ have made the rapid collection of individual body size data feasible. A recent study of assemblage-level microfossil size and shape presented and utilized technological advances in imaging and novel open-source software, *AutoMorph*, to generate specimen-level data including individual images, 3D hulls, and 2D and 3D morphometric data using photogrammetry^14^. In this study, we used a modified version of *AutoMorph* that includes image processing capacities for macroscopic photographic images in order to document and measure modern Patellogastropoda from Baja California, Mexico to Alaska in the University of California Museum of Paleontology (UCMP) collections. The resulting images are tagged with metadata and associated with specimen-level morphometric measurements. All morphological information, images and metadata are openly available through Zenodo (Data Citation 1). The physical specimens associated with these images are reposited at the UCMP.

**Figure 1:**
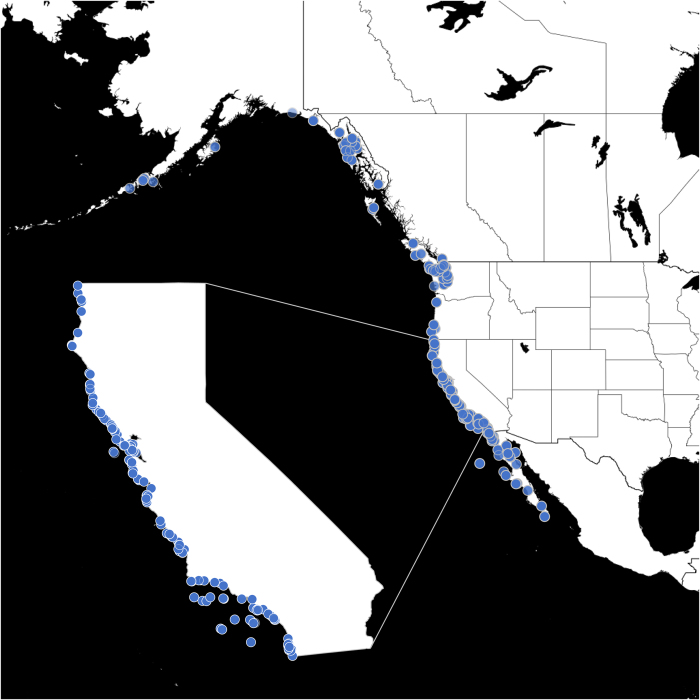
Map of northeastern Pacific with sample sites. A map of the area of the northeastern Pacific referenced in this study, with sample localities in blue. California sites are further highlighted, as the majority of localities (216 of 355) from which specimens were collected were within California.

Collection localities ranged from 60°06′11N (Cape Yakataga, Alaska, United States) to 22°89′56N (Cabo San Lucas, Baja California, Mexico) (Fig. 1 and Table 1 (available online only)). The northeastern Pacific was chosen because the UCMP has extensive patellogastropod collections from this region and because mean annual temperatures, seasonality, and productivity exhibit strong variation over ~5,000 km of coastline^15^. 1,410 lots of recent northeastern Pacific patellogastropod specimens from the UCMP collections were imaged in the summer of 2015 by S.S.K. Of these, 1,256 lots were successfully processed via *AutoMorph* for the morphometric parameters reported here. Most processing failures for the 155 unsuccessful lots resulted from inadequate focusing of stacked images during the *AutoMorph* step (*focus*) that required use of a third-party stacking software. Each lot contained between 1 and 223 individuals (Table 1 (available online only)), and were collected between 1860–2014 by numerous collectors for a variety of reasons (e.g., taxonomic, distributional, ecological).

We present images and morphometric data for 12,035 individual patellogastropods, representing 30 species from 355 northeastern Pacific sites (Tables 2 and 3 (available online only)). This is, to our knowledge, the largest assemblage-scale morphometric dataset ever produced for mollusks, and documents northeastern Pacific patellogastropod size and diversity over the past 150 years.

## Methods

### Sampling

More than 90% of recent northeastern Pacific patellogastropod collections from the UCMP were photographed for this study. Because specimens in the collection had been identified to the species level and grouped by locality during accession to the museum, each lot reported in Table 1 (available online only) represents a single collection event of a single species at a single site. For this study, 1,256 of these lots from 355 unique localities between 60°06′11N and 22°89′56N (Table 2 (available online only)) were photographed and measured via digital image processing. A large number of these lots (699) were uncurated, and were assigned UCMP lot numbers prior to incorporation in the study. This preliminary curation resulted in the digitization of previously unavailable collection information (Table 1 (available online only)). Locality information was recorded as both UCMP locality numbers (e.g., ‘D8919’) and locality strings (e.g., ‘Monterey, CA’). It should be noted that multiple UCMP locality numbers may be assigned to a single site, as samples are assigned numbers at the time of accession that reflect both the locality as well as the accession group. Locality strings were matched to latitudinal and longitudinal coordinates using GEOLocate^16^ for all but 40 lots, and coordinates were rounded to the nearest 0.1 degree to account for potential measurement uncertainty (Table 1 (available online only)).

The UCMP recent mollusk collections date from the Geological Survey of California (1860–1874) and were deposited by the State Legislature with the university in 1873 (ref. 17). J.G. Cooper was especially active in the building of the recent mollusk components of these collections^18^. The early survey collections were further augmented by late 19th and early 20th century collections from Henry Hemphill (1830–1914)^19^ and Josiah Keep (1849–1911)^20^. Additional large northeastern Pacific collections were added in the mid 20th century with the acquisition of the Eugene Coan and Rudolph Stohler mollusk collections from the University of California Davis^21^. In addition to general mollusk collections, collections focused exclusively on patellogastropods (Gulf of California through Alaska) were made as part of faculty and graduate research projects, including those of Avery Ransome Grant Test^22^ (1933–1945), Henry K. Fritchman^23^ (1950s), and David R. Lindberg and students (1982–2014). These collection activities over the last 150+ years produced the systematic collection of northeastern Pacific patellogastropods reported on here.

### High-throughput imaging

**Figure 2:**
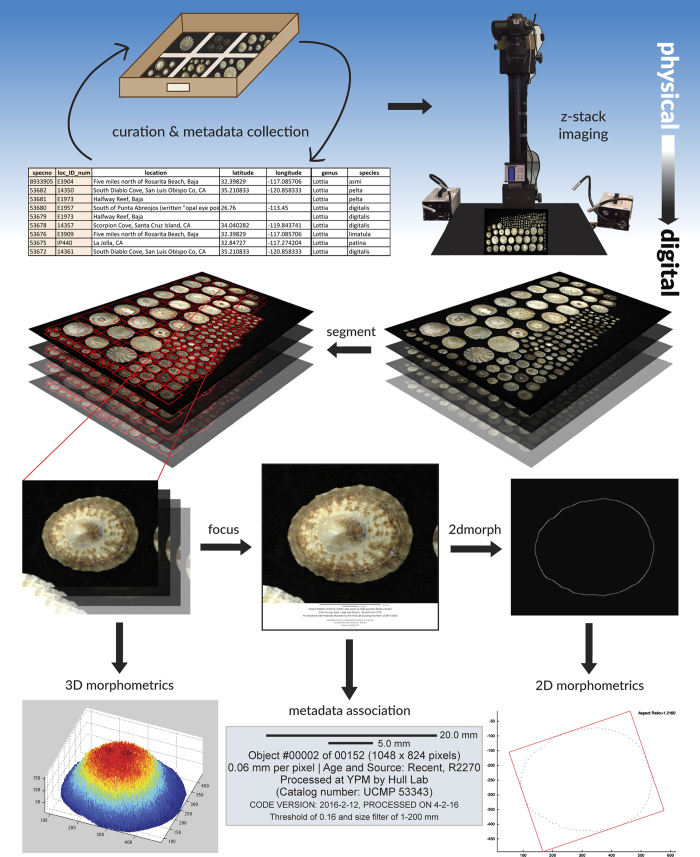
Digitization workflow overview. All physical specimens were curated and their metadata catalogued prior to imaging. Specimen lots were imaged in Z-stacks (Row 1). Following digitization, individual objects were identified from these lot images, and each object was cut out of the Z-stack and labeled with metadata using the *segment* module of *AutoMorph* (Row 2). These Z-stack images were then passed through the *focus* module to create 2D EDF images for each individual object, and metadata labels were again associated with each individual’s 2D EDF (Row 3). An enlarged example of this image-associated metadata is provided (Row 4, center). Following the *focus* step, 2D EDFs were passed through the *run2dmorph* module to generate 2D morphometric data (Rows 3 and 4) while object Z-stacks were retained for future 3D morphometric work (Row 4).

Images were taken using a Canon EOS 5D Mark III camera and a Canon EF 100 mm f/2.8 Macro USM lens. Camera settings were manually optimized for macro imaging: an aperture of 2.8 and ISO of 200 were used for all images. Image settings and file storage were remotely controlled by a laptop computer using the Canon EOS Utility 3 program. The camera was mounted on a Cognysis Inc. Stackshot Automated Focus Stacking Rail, which in turn was mounted on a camera stand (Fig. 2). StackShot was used to standardize the distance between image planes. The Z-stack images generated by StackShot allowed for the creation of 2D extended-depth-of-focus images and 3D photogammetry^13^ (for use in future studies). The distance between each z-plane was set to 1 mm per step. The number of planes varied according to the maximum height of the tallest specimen in each sample. Samples were illuminated during imaging using standard gooseneck illuminators (Fig. 2). Use of these illuminators resulted in color alteration, which was corrected during imaging using the Canon EOS Utility software.

High-throughput imaging techniques, modelled after previous studies on foraminifera^13,14^, were utilized to maximize the efficiency of digitization. Samples were imaged as sample lots as opposed to individual specimens, with a maximum of 150 individuals per round of photography. Specimens were laid out on a uniformly dark background such that no individuals were touching. Prior to each round of digitization, scale bars were photographed in X and Y directions in order to calibrate the image scale in mm/pixel for post-processing.

### *AutoMorph*: Automated morphometric post-processing

We utilized the Yale Grace high-performance computing cluster to expedite image post-processing with the *AutoMorph* software suite (available on GitHub; see *Code Availability*). *AutoMorph*^14^ is a software package that utilizes photographic image stacks of multiple objects to extract individual object images and associated morphometric measurements and coordinates. Some aspects of the *AutoMorph* software are specific to macroscopic images and we refer to them here as the macro-version *AutoMorph*.

#### Image preparation

The macro-version *AutoMorph* contains a software module, *prepare*, which ensures that any frame shifting associated with camera zooming in the Z-dimension is eliminated. Using *prepare*, all image stacks were rotated and scaled prior to processing with *segment*.

#### Segmenting

**Figure 3:**
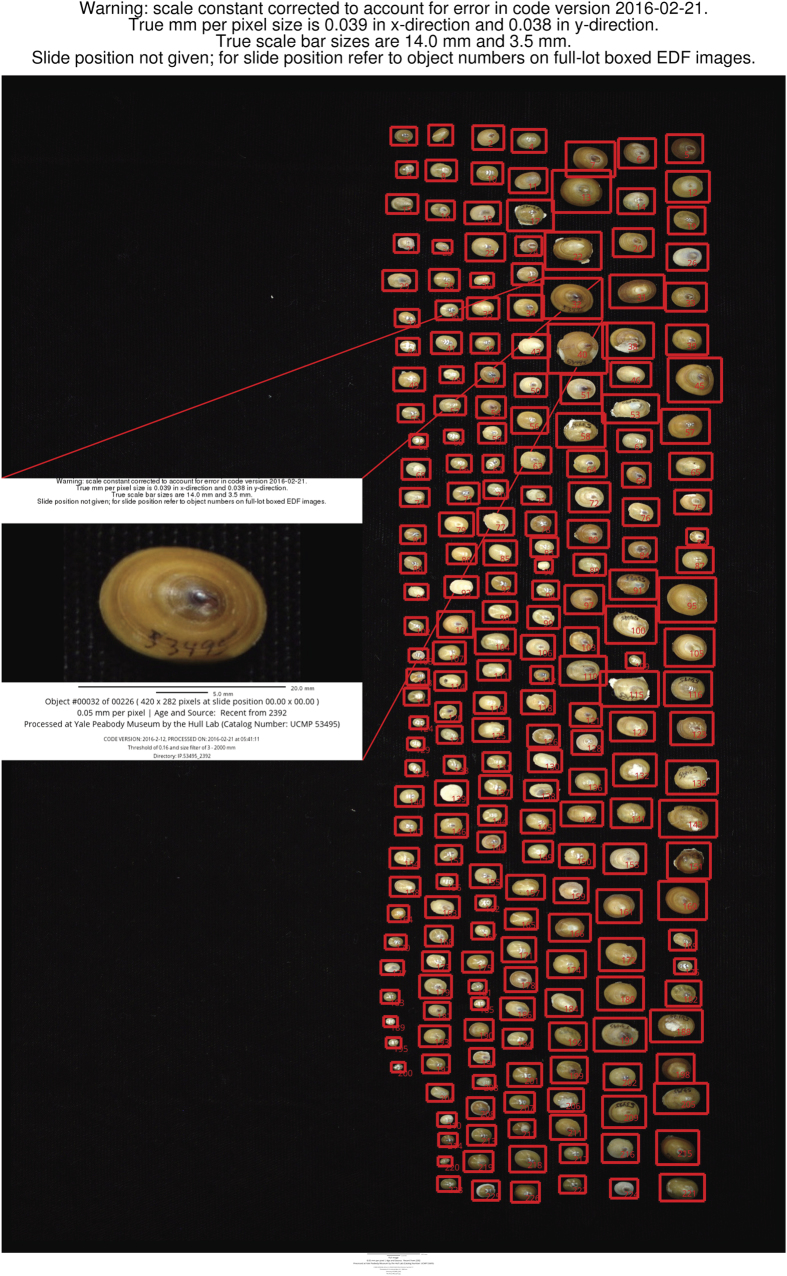
Example labeled image. An example overview (background) and individual image (inset). Objects are boxed and labeled with their respective object numbers, which are then used to identify individual images. Both overview and individual images are tagged with associated metadata. The images described here contain additional warning labels that provide users with corrected metadata (i.e., scaling constant and, when applicable, locality information). Shown here: UCMP 53,495 (*Lottia insessa, N*=223), with the EDF of object #32 inset.

Image stacks of individual patellogastropod limpets were extracted from full-lot images using the *AutoMorph* module *segment*^14^. *segment* takes advantage of the color contrast between objects and the image background to identify and box each individual. These objects are then cut out of the full-lot image stack and placed into individual directories containing object-specific z-sliced images. Object numbers are assigned during processing and are labeled on both the full lot image and the individual stacks. User-defined metadata is also attached to each image by *segment*, including scale bars and processing date and location (Fig. 3).

#### Focusing

Image stacks were processed into a best 2D image, known as an extended-depth-of-focus (EDF) image, using the *AutoMorph* package *focus*^14^. *focus* facilitates batch processing in *Zerene Stacker* (http://zerenesystems.com/cms/stacker), a third-party focus stacking software, or ImageJ, an open source image processing toolkit. *focus* retains individual image z-stacks for downstream use in 3D data extraction using *run3dmorph*^13,14^, another *AutoMorph* module.

#### 2D morphometrics

EDF images for each object were then processed to extract object outlines and 2D morphometric measurements using the *AutoMorph* package *run2dmorph*^14^. This 2D morphometric software passes images through smoothing, RGB, greyscale, and black-and-white filters to extract object outlines, *x,y*-coordinates, and individual measurements of size and shape, such as major axis length, minor axis length, area, perimeter, rugosity, and aspect ratio. These measurements are saved in sample-specific CSV files, and object outlines are plotted on their respective images to allow for visual quality-control checks of 2D data extraction.

### Code availability

Both micro- and macro-specific versions of the *AutoMorph* software suite are freely accessible on GitHub (https://www.github.com/HullLab/AutoMorph). This study used *AutoMorph* v2016-02, the first macro-specific version of the code. Since completion of our dataset, *AutoMorph* has been further updated to remove scaling errors (see below) and allow for macro-specific 3D processing.

## Data Records

Individual metadata, images, and shape data are provided both in this data report and on the Zenodo data repository (Data Citation 1). The tables in this data report provide relevant metadata including UCMP specimen and locality numbers, locality information, and taxonomy; summary statistics; and information used in technical validation of measurements. Table 1 (available online only) details UCMP specimen numbers and locality numbers, site names, rounded coordinates, and the number of individuals per lot successfully extracted for 2D shape. Each unique site is listed with all associated localities in Table 2 (available online only) along with the number and names of species at that site. Table 3 (available online only) lists the latitudinal range minima and maxima for each species, as well as the UCMP specimen numbers associated with each site and the total number of individuals measured per species. Table 4 (available online only) provides a comparison of *AutoMorph-* and hand-generated measurements. Table 5 (available online only) details the few instances of locality information mismatches present between the UCMP online database and specimen-associated locality information (see *Usage Notes*). Supplementary Table 1 provides a list of all objects included in the dataset, including an indication of which objects have known locality coordinates.

The data reported on here are all available on Zenodo under a single citation (Kahanamoku *et al.* 2017) and include:

1. boxed_full_images.zip: 1,256 full-lot (overview) images with boxed object identified by the *segment* module.
2. zstacks.zip: image stacks for each of the 1,256 lots processed by *segment*. These image stacks may potentially be used to generate individual 3D height maps.
3. 2d_edfs_labeled.zip: 12,035 individual EDF images produced by *focus*. A list of the individuals represented in the dataset are provided in Supplementary Table 1 in metadata.zip.
4. 2d_outlines.zip: 12,035 EDF images with 2D outline overlays for quality control.
5. 2d_coordinates.zip: csv files of the outline shape coordinates for the 1,256 lots successfully processed with *AutoMorph*, including one file (all_coordinates_limpets.csv) with the coordinates for all 12,035 individuals extracted.
6. master_2d_measurements.csv: UCMP locality numbers, UCMP lot numbers, locality coordinates, object identification numbers output by *AutoMorph*, and morphometric data for all 12,035 individuals. Each individual has associated full-lot, individual EDF, 2D and outline images, as well as outline shape coordinates, all of which may be found in the zip files listed above. Object identification numbers, which are used to link morphometric measurements with specific individuals, can be found in the column ‘object IDs’. Morphometric data provided includes major axis length, minor axis length, perimeter length, eccentricity, and rugosity, as well as aspect ratio and the height and width of the aspect rectangle. Locality coordinates are rounded to the nearest 0.1 degree to account for potential measurement uncertainty.
7. metadata.zip: A directory containing the tables presented in this publication (Tables 1–5 (available online only)) and Supplementary Table 1) in corresponding.xslx files.

The first data product, boxed_files.zip, is also available on the UCMP’s photography database (http://calphotos.berkeley.edu/). The museum-specific metadata in Table 1 (available online only) can also be found using the UCMP specimen and locality database (http://ucmpdb.berkeley.edu/).

## Technical Validation

### Size validation

**Figure 4:**
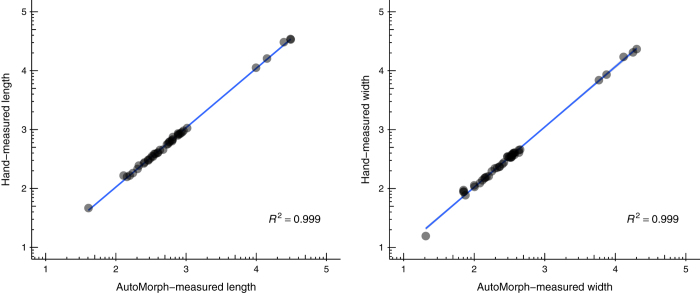
Size validation. A comparison of *AutoMorph*- and hand-measured length and width (data in Table 4 (available online only)). Length R^2^: 0.99; width R^2^: 0.99; *n*=41.

The lengths and widths of 41 limpets measured by hand were compared to *AutoMorph* outputs of size (Fig. 4, Table 4 (available online only)). *AutoMorph* measurements correspond well to hand measurements of length (*P*<2.2×10^16^; intercept −0.53) and width (*P*<2.2×10^16^; intercept −0.56), with an R^2^ of 0.999 for both. The largest difference between *AutoMorph* and hand measurements was 7.6 mm, while the mean difference was 0.83 mm. S.d. of the residuals was approximately 0.6 mm for both length and width—well within the variability produced by traditional measurements^24^. We refer interested readers to the Supplementary Text of Hsiang *et al.*^14^, which includes a further comparison between *AutoMorph* and hand measurements for ~100 fossil patellogastropods.

### Image warning labels

*AutoMorph* v2016-02 contained a scaling error that resulted in image scale bars that were consistently 70% larger than true size. The scale bars on all image products from this study are, as a result, 70% too long. Images were appended with warning labels to alert users of the error in scale bar calculation (Fig. 3). The correct pixel size was scaled by a factor of 10 in the 2D image processing steps in order to produce larger output images for visual outline checks. The 2D measurements resulting from this image processing step were subsequently corrected, such that data products 4 and 5 (2D outline coordinates and 2D measurements) are correct as reported.

### Object selection with *segment*

The *AutoMorph* module *segment* produces overview images with each individual object boxed in red (Fig. 3; full set of boxed images available in boxed_full_images.zip in data citation). To verify that all objects were being selected for segmenting, the module was first run in ‘sample’ mode, and image selection parameters were visually optimized to allow for proper segmentation.

### Shape extraction with *run2dmorph*

2D extended-depth-of-focus (EDF) images produced by the *focus* module were visually inspected to ensure that the images were properly composited. These EDFs were then passed through the *run2dmorph* module to generate 2D shape and outline extraction. The quality of 2D shape extraction was visually checked for each object using outline-object overlays (see ‘2d_outlines.zip’ in data citation). Parameters in *run2dmorph* were adjusted as necessary to optimize the fidelity of the 2D outlines extracted.

## Usage Notes

### Expected collection biases

**Figure 5:**
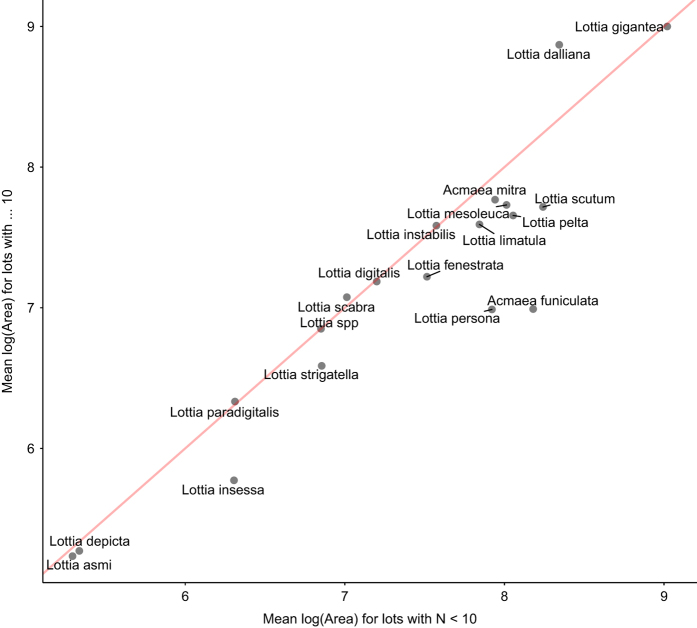
Collection biases in lots with small sample sizes. Lots with small sample sizes (defined here as lots with less than 10 individuals) may make collector biases towards larger body size more apparent. Species mean body size for small lots (*N*<10) is compared with mean body size for large lots (*N*>10); while some species means lie along the line of unity, the majority (12 of 19) appear to have larger mean body sizes when lot sample sizes are small.

Collection biases are likely to present themselves in lots with small sample sizes, as small lots often introduce collector biases towards larger body sizes (Fig. 5). Users may want to systematically cull the dataset (i.e., perform lot size stepping) in order to account for biases introduced as a result of different collection purposes.

### Curation of locality information

All specimen lots were assigned UCMP locality numbers upon accession into the collection. For three sample lots, the locality information physically associated with the specimens did not match locality information in the online UCMP database. For these sample lots, specimen-associated locality information was considered primary and thus included in the dataset. A record of the mismatched coordinates can be found in Table 5 (available online only).

A number of localities had no associated GPS coordinates, or their locality information was too vague to be associated with GPS coordinates using GEOLocate^16^. Individuals collected from these localities remain in the database but their corresponding locality information was collected directly from specimen-associated catalogue cards and may not be correct.

## Additional information

**How to cite this article:** Kahanamoku, S. S. *et al.* Twelve thousand recent patellogastropods from a northeastern Pacific latitudinal gradient. *Sci. Data* 5:170197 doi: 10.1038/sdata.2017.197 (2018).

**Publisher’s note:** Springer Nature remains neutral with regard to jurisdictional claims in published maps and institutional affiliations.

## Supplementary Material



Supplementary Table S1

## Figures and Tables

**Table 1:** UCMP specimen and locality numbers, site names, rounded coordinates, number of individuals per lot

**UCMP specimen number**	**UCMP locality number**	**locality string**	**latitude**	**longitude**	**number of individuals**
53000	E844	Point Fermin, CA	33.7	−118.3	1
53001	IP16029	Horseshoe Cove, CA	38.3	−123.1	9
53002	A4138	Baja	NA	NA	1
53003	2782-	San Hippolita Point, Baja	48.6	−123.2	17
53005	E3114	Punta Piedra, Baja	32.1	−116.9	2
53006	E377	Government Point, CA	34.4	−120.5	4
53007	2391-	Santa Barbara Island, CA	33.5	−119	1
53008	2403-	Santa Cruz, CA	37	−122	22
53009	E4025	San Quentin Bay, CA	37.9	−122.5	2
53010	E139	Point Pinos, Pacific Grove, CA	36.6	−121.9	7
53011	E2992	Solana Beach, Del Mar, CA	33	−117.3	1
53013	E518	San Simeon, CA	35.6	−121.2	5
53016	2786-	San Simeon Point, CA	35.6	−121.2	35
53017	E3251	La Mision River, Baja	32.1	−116.9	20
53018	2388-	San Pedro, CA	33.7	−118.3	3
53019	B835	Coronado, CA	32.7	−117.2	45
53020	2390-	Santa Barbara, CA	34.4	−119.7	1
53021	2409-	La Jolla, CA	32.8	−117.3	5
53022	3117-	Punta Piedra, Baja	32.1	−116.9	7
53023	E3167	Dana Point, CA	33.5	−117.7	8
53024	2898-	Fort Point, CA	37.8	−122.5	2
53025	E679	Whites Point, San Pedro Qd, CA	33.7	−118.3	6
53026	R1754	Tomales Bay, CA	38.2	−122.9	8
53027	2393-	San Francisco, CA	37.8	−122.4	2
53028	2400-	Puget Sound, WA	47.8	−122.4	3
53029	B829	Mission Bay, CA	32.8	−117.2	16
53030	E4950	Estero de Punta Banda, Baja	31.7	−116.7	2
53031	E511	Moonstone Beach, Cambria, CA	35.6	−121.1	6
53033	R5048	Mexico	NA	NA	3
53034	B829	Mission Bay, CA	32.8	−117.2	3
53036	E1964	Rio Guadalupe, Baja	32	−116.5	5
53037	2392-	San Diego, CA	32.7	−117.2	3
53038	B829	Mission Bay Breakwater, San Diego, CA	32.8	−117.2	4
53039	2408-	Laguna Bay, CA	35.3	−120.7	9
53040	2411-	Pigeon Point, CA	37.2	−122.4	2
53041	2403-	Santa Cruz, CA	37	−122	2
53042	R1688	San Diego Jetty, San Diego, CA	32.7	−117.2	1
53043	2392-	San Diego, CA	32.7	−117.2	32
53044	E6873	Punta Banda, Baja	31.7	−116.7	1
53045	2388-	San Pedro, CA	33.7	−118.3	89
53046	2392-	San Diego, CA	32.7	−117.2	121
53047	2435-	Lagoon Head, Baja	NA	NA	11
53048	2773-	Tomales Bay, CA	38.2	−122.9	47
53049	E481	Palos Verdes Point, Redondo Beach, CA	33.8	−118.4	36
53050	D1915	Isla Guadalupe, Baja	29	−118.3	5
53051	E3748	Punta Banda, Baja	31.7	−116.7	7
53052	2910-	Port Harford, CA	35.2	−120.8	4
53053	A3991	Newport Beach, CA	33.6	−117.9	7
53054	2778-	San Clemente Island, CA	32.9	−118.5	1
53055	IP14536	Halfway Reef Cliffs, Baja	NA	NA	4
53056	E6870	San Diego, CA	32.7	−117.2	1
53057	R1688	San Diego Jetty, San Diego, CA	32.7	−117.2	100
53058	2392-	San Diego, CA	32.7	−117.2	77
53059	IP14596	Sunset Cliffs, San Diego, CA	32.7	−117.3	1
53060	IP14592	San Diego, CA	32.7	−117.2	7
53061	IP14597	Point Loma, CA	32.7	−117.2	1
53062	E3749	Punta Banda, Baja	31.7	−116.7	22
53063	D8930	San Diego, CA	32.7	−117.2	1
53064	D8930	San Diego, CA	32.7	−117.2	3
53065	D8930	San Diego, CA	32.7	−117.2	4
53066	D8930	San Diego, CA	32.7	−117.2	7
53067	IP14364	Intake Cove, Diablo Canyon Power Plant, CA	35.2	−120.9	1
53068	R516	Bear Bay, Baranoff Island, AK	57.5	−135.5	4
53069	D8919	Half Moon Bay, CA	37.5	−122.4	3
53070	E6872	Morro Rock, CA	35.4	−120.9	7
53071	D8919	Monterey, CA	36.6	−121.9	2
53072	D8919	Monterey, CA	36.6	−121.9	2
53073	D8919	Monterey, CA	36.6	−121.9	5
53074	E696	Morro Rock, CA	35.4	−120.9	2
53075	E527	Willows Anchorage, Santa Cruz Island, CA	34	−119.8	10
53077	E998	Northwest Mission Bay, San Diego, CA	32.8	−117.2	3
53078	E1402	Monarch Bay, CA	33.5	−117.7	14
53079	E757	Santa Cruz, CA	37	−122	2
53080	E3836	Half mile north of Point Buchon, CA	35.3	−120.9	2
53081	D8919	Tomales Bay, CA	38.2	−122.9	7
53083	E2319	North Cedros Island, Baja	28.1	−115.2	3
53084	E827	Yankee Point, Soberones, CA	36.5	−121.9	3
53085	E703	Cayucos, CA	35.4	−120.9	7
53086	E782	Soberanes Point, CA	36.4	−121.9	3
53087	E3001	Bird Rock, CA	32.8	−117.3	4
53088	E6875	Ensenada, Baja	31.9	−116.6	1
53089	E564	Santa Catalina Island, CA	33.4	−118.4	7
53090	E5	Whaling Station, Point Lobos Reserve, Monterey, CA	36.5	−122	7
53091	D9260	Punta Banda, Baja	31.7	−116.7	5
53092	E443	Dana Point, CA	33.5	−117.7	14
53093	E581	Santa Barbara Island, CA	33.5	−119	2
53094	E4083	Anaheim Landing, CA	33.7	−118.1	6
53095	2434-	Bahia Tortugas, Baja	27.7	−114.9	57
53097	E6871	Venice, CA	34	−118.5	9
53099	E155	Tomales Point, CA	38.2	−123	1
53100	E99	Moss Beach, Half Moon Bay, CA	37.5	−122.5	1
53101	E1662	Newport Harbor, Newport Beach, CA	33.6	−117.9	8
53102	E613	San Miguel Island, Santa Barbara, CA	34	−120.4	8
53105	2778-	San Clemente Island, CA	32.9	−118.5	2
53107	A4198	Baja	NA	NA	19
53108	E6869	San Simeon, CA	35.6	−121.2	2
53109	E6874	Anaheim Bay, CA	33.7	−118.1	4
53110	E6868	Cabrillo Beach, CA	33.7	−118.3	43
53111	E540	Santa Rosa Island, Santa Barbara, CA	34	−120.1	10
53112	E244	One mile south of Hardy, CA	39.7	−123.8	1
53113	E375	Government Point, CA	34.4	−120.5	24
53114	E445	Three miles east of Gaviota, CA	34.5	−120.2	16
53115	E9937	San Pedro, CA	33.7	−118.3	6
53116	R7162	Twin Rivers, WA	48.2	−124	1
53117	A3991	Newport Beach, CA	33.6	−117.9	3
53118	2393-	San Francisco, CA	37.8	−122.4	23
53119	B835	Coronado, CA	32.7	−117.2	28
53120	2432-	Port Townsend, WA	48.1	−122.8	9
53121	2400-	Puget Sound, WA	47.8	−122.4	2
53122	E38	Duxbury Reef, CA	37.9	−122.7	12
53123	E843	Point Fermin, CA	33.7	−118.3	2
53124	E1579	Arena Cove, CA	38.9	−123.7	25
53125	E6847	Port Orford, OR	42.7	−124.5	49
53126	R4093	Beaver Cove, Vancouver Island, BC	50.5	−126.9	2
53128	2389-	Santa Catalina Island, CA	33.4	−118.4	7
53129	2410-	Half Moon Bay, CA	37.5	−122.4	14
53130	2889-	Point Arena, CA	38.9	−123.7	3
53131	D8930	San Diego, CA	32.7	−117.2	4
53132	E526	Willows Anchorage, Santa Cruz Island, CA	34	−119.8	31
53133	E259	Near mouth of Elk Creek, South of Elk Creek, CA	39.1	−123.7	11
53134	E803	Fort Bragg Landing Beach, CA	39.4	−123.8	18
53135	2408-	Laguna Bay, CA	35.3	−120.7	20
53136	2787-	Fort Bragg, CA	39.4	−123.8	8
53137	2409-	La Jolla, CA	32.8	−117.3	7
53138	2402-	AK	NA	NA	4
53139	R7174	Restoration Point, Bainbridge Island, WA	47.6	−122.5	3
53140	2436-	Golden Gate, CA	37.8	−122.5	43
53141	2435-	Lagoon Head, Baja	NA	NA	7
53142	2440-	Tobin Beach, CA	NA	NA	4
53143	E367	One mile north of Morro Bay, CA	35.4	−120.9	9
53144	E557	Pelican Bay, Santa Cruz Island, CA	34	−119.8	3
53145	2881-	San Miguel Island, Santa Barbara, CA	34	−120.4	12
53146	E611	South end of Cuyler Harbor, San Miguel Island, CA	34	−120.4	2
53147	E458	Dana Point, CA	33.5	−117.7	10
53148	2433-	San Nicolas Island, CA	33.2	−119.5	48
53149	2891-	Stewart's Point, CA	38.7	−123.4	6
53150	2786-	San Simeon Point, CA	35.6	−121.2	9
53151	R521	Hoonah, Chichagoff Island, AK	58.1	−135.4	4
53152	2780-	Santa Cruz Island, CA	34	−119.8	29
53153	D8930	San Diego, CA	32.7	−117.2	1
53154	2890-	Seattle, WA	47.6	−122.3	4
53155	E3750	North side of Punta Banda, Baja	31.7	−116.7	6
53156	2785-	Russian Gulch, CA	39.3	−123.8	10
53158	2395-	Monterey, CA	36.6	−121.9	5
53159	2776-	Santo Tomas, Baja	31.6	−116.4	7
53160	2395-	Monterey, CA	36.6	−121.9	10
53161	2895-	Morro Bay, CA	35.4	−120.8	1
53162	2430-	Chatham Sound, Queen Charlotte Island, BC	53.3	−132.1	4
53163	E3113	Punta Piedra, Baja	32.1	−116.9	8
53164	2779-	Port Angeles, WA	48.1	−123.4	31
53165	2778-	San Clemente Island, CA	32.9	−118.5	2
53166	D8930	San Diego, CA	32.7	−117.2	4
53167	2496-	Humboldt County, CA	40.7	−124.2	2
53168	E1279	Anacortes, WA	48.5	−122.6	4
53169	E1400	Half mile north of Dana Point, CA	33.5	−117.7	5
53170	E859	Stewart's Point, CA	38.7	−123.4	6
53171	E36	Richmond Point, CA	37.9	−122.4	1
53172	E138	Point Pinos, Pacific Grove, CA	36.6	−121.9	19
53173	R1685	Golden Gate, CA	37.8	−122.5	36
53174	2398-	Straits of Fuca, Vancouver Island, CA	48.3	−124	3
53175	2773-	Tomales Bay, CA	38.2	−122.9	11
53176	E223	Two miles south of Cape Mendocino, CA	40.4	−124.4	3
53177	R5030	Point Lobos, CA	36.5	−122	6
53178	IP14349	Intake Cove, Diablo Canyon Power Plant, CA	35.2	−120.9	2
53179	E3059	Punta Banda, Baja	31.7	−116.7	6
53180	E695	Morro Rock, CA	35.4	−120.9	5
53181	E826	Yankee Point, Soberones, CA	36.5	−121.9	22
53182	E3250	South of La Mision River, Baja	32.1	−116.9	8
53183	2394-	San Francisco Bay, CA	37.7	−122.3	2
53184	2428-	Coos Bay, OR	43.4	−124.2	15
53185	R1698	Monterey, CA	36.6	−121.9	16
53186	IP439	Del Mar Point, CA	38.7	−123.5	4
53187	IP10951	Davenport, CA	37	−122.2	4
53188	IP10590	Tatoosh Island, WA	48.4	−124.7	1
53189	2419-	Moss Beach, Half Moon Bay, CA	37.5	−122.5	1
53190	IP14356	Diablo Rock, CA	35.2	−120.9	1
53191	E865	Near Hare Creek, south of Fort Bragg, CA	39.4	−123.8	2
53192	E573	South Anacapa Island, Ventura, CA	34	−119.4	27
53193	E1260	Del Mar Ranch, CA	38.7	−123.5	12
53194	D8919	Moss Beach, Half Moon Bay, CA	37.5	−122.5	3
53195	D8919	Carmel Point, CA	36.5	−121.9	9
53196	IP10590	Tatoosh Island, WA	48.4	−124.7	21
53197	2395-	Monterey, CA	36.6	−121.9	24
53199	2395-	Monterey, CA	36.6	−121.9	46
53200	2403-	Santa Cruz, CA	37	−122	3
53201	2770-	San Juan Island, WA	48.5	−123.1	1
53202	E136	Moss Beach, Half Moon Bay, CA	37.5	−122.5	3
53203	2432-	Port Townsend, WA	48.1	−122.8	1
53204	2434-	Bahia Tortugas, Baja	27.7	−114.9	9
53205	2785-	Russian Gulch, CA	39.3	−123.8	1
53206	E1824	Point Loma, CA	32.7	−117.2	2
53207	E1800	Point Loma, CA	32.7	−117.2	1
53208	B7361	Chatham, AK	57.5	−134.9	3
53209	IP10581	Tatoosh Island, WA	48.4	−124.7	3
53210	E6891	Piedras Blancas, CA	35.7	−121.3	3
53211	E6888	Pigeon Point, CA	37.2	−122.4	8
53212	E6889	Moss Beach, Half Moon Bay, CA	37.5	−122.5	6
53213	2787-	Fort Bragg, CA	39.4	−123.8	2
53214	2779-	Port Angeles, WA	48.1	−123.4	1
53215	R3445	Point Loma, CA	32.7	−117.2	3
53216	E1847	Mouth of Tijuana River, Imperial Beach, CA	32.6	−117.1	3
53217	IP14595	Point Loma Lighthouse, CA	32.7	−117.2	1
53218	2418-	Cape Mendocino, CA	40.4	−124.4	2
53219	2388-	San Pedro, CA	33.7	−118.3	1
53220	IP10581	Tatoosh Island, WA	48.4	−124.7	1
53221	E512	Moonstone Beach, Cambria, CA	35.6	−121.1	1
53222	2392-	San Diego, CA	32.7	−117.2	65
53223	E737	Shell Beach, Bodega Head, CA	38.4	−123.1	12
53224	IP445	Mussel Point, CA	38.3	−123.1	1
53225	2436-	Golden Gate, CA	37.8	−122.5	28
53226	2395-	Monterey, CA	36.6	−121.9	2
53227	B835	Coronado, CA	32.7	−117.2	3
53228	E6905	Venice, CA	34	−118.5	57
53229	R1688	San Diego Jetty, San Diego, CA	32.7	−117.2	29
53230	2395-	Monterey, CA	36.6	−121.9	7
53231	IP14364	Intake Cove, Diablo Canyon Power Plant, CA	35.2	−120.9	1
53232	E582	Off of the south-southeast shore of Santa Barbara Island, CA	33.5	−119	1
53233	E773	Mussel Point, CA	38.3	−123.1	1
53234	E459	Dana Point, CA	33.5	−117.7	1
53236	E390	One mile north of San Simeon, CA	35.6	−121.2	18
53237	E783	Soberanes Point, CA	36.4	−121.9	3
53238	E845	Point Fermin, CA	33.7	−118.3	23
53239	E1263	Del Mar Ranch, CA	38.7	−123.5	13
53240	E6904	Arch Rock, CA	38	−122.8	12
53241	E6913	Piedras Blancas Point, CA	35.7	−121.3	3
53242	2395-	Monterey, CA	36.6	−121.9	3
53243	E667	Near mouth of Scott Creek, Davenport, CA	37	−122.2	11
53244	E759	Santa Cruz, CA	37	−122	5
53245	E615	San Miguel Island, Santa Barbara, CA	34	−120.4	4
53246	E857	Bodega Head, CA	38.3	−123.1	1
53247	E508	Moss Landing, CA	36.8	−121.8	10
53249	E199	Near CA-OR border, Smith River, CA	41.9	−124.2	5
53250	E541	Santa Rosa Island, Santa Barbara, CA	34	−120.1	2
53253	IP447	Puget Sound, WA	47.8	−122.4	1
53254	E689	Gaviota Beach, CA	34.5	−120.2	5
53256	E51	Dillon Beach, CA	38.3	−123	3
53257	E796	Goat Rock, CA	38.4	−123.1	3
53258	E594	San Nicolas Island, CA	33.2	−119.5	5
53260	E559	Pelican Bay, Santa Cruz Island, CA	34	−119.8	2
53262	E537	Garedon Canyon, Santa Rosa Island, CA	34	−120.1	1
53278	E179	Russian Gulch, CA	39.3	−123.8	2
53280	2392-	San Diego, CA	32.7	−117.2	3
53290	D8915	Neah Bay, WA	48.4	−124.6	4
53291	E6918	Crescent Beach, WA	48.7	−122.9	10
53292	R517	Red Bluff Bay, Baranoff Island, AK	56.9	−134.8	2
53293	R519	Windfall Harbor, Admiralty Island, AK	57.9	−134.3	4
53294	2402-	AK	NA	NA	1
53295	B3562	Southwest AK	NA	NA	19
53299	R7111	British Columbia	NA	NA	7
53301	3394-	Victoria, BC	48.4	−123.4	9
53302	IP437	Southeast corner of San Nicolas Island, CA	33.2	−119.5	14
53303	E6903	Cabrillo Beach, CA	33.7	−118.3	77
53304	R521	Hoonah, Chichagoff Island, AK	58.1	−135.4	3
53306	R519	Windfall Harbor, Admiralty Island, AK	57.9	−134.3	2
53309	R5044	Yakutat, AK	59.5	−139.7	2
53310	R518	Glacier Bay, AK	58.8	−136.3	3
53311	IP14597	Point Loma Lighthouse, CA	32.7	−117.2	1
53312	IP14597	Point Loma Lighthouse, CA	32.7	−117.2	3
53313	D8919	Crescent City, CA	41.8	−124.2	2
53315	D9261	Blowhole west of Bauda Fish Camp, Baja	31.7	−116.7	4
53316	R519	Windfall Harbor, Admiralty Island, AK	57.9	−134.3	6
53317	IP448	North Jelly Mid Tidal Zone, Humboldt Bay, CA	40.7	−124.2	3
53319	B7363	Chatham, AK	57.5	−134.9	1
53320	R4093	Beaver Cove, Vancouver Island, BC	50.5	−126.9	2
53321	IP14594	Point Loma Lighthouse, CA	32.7	−117.2	2
53322	E860	Stewart's Point, CA	38.7	−123.4	1
53323	D8919	Moss Beach, Half Moon Bay, CA	37.5	−122.5	5
53324	D8919	Monterey, CA	36.6	−121.9	2
53325	D8919	Monterey, CA	36.6	−121.9	4
53326	D9261	Blowhole west of Bauda Fish Camp, Baja	31.7	−116.7	4
53327	D8919	Moss Beach, Half Moon Bay, CA	37.5	−122.5	14
53328	D8919	Monterey, CA	36.6	−121.9	6
53329	3122-	Bridgeport, OR	43	−124	2
53330	2428-	Coos Bay, OR	43.4	−124.2	15
53331	E447	Gaviota Beach, CA	34.5	−120.2	4
53332	E2218	Rock Point, Point Bonita, CA	37.8	−122.5	1
53333	2888-	Port Crescent, WA	48.2	−123.7	19
53334	2779-	Port Angeles, WA	48.1	−123.4	35
53335	R3124	WA	NA	NA	8
53336	R7209	Pillar Point, WA	48.2	−124.1	3
53337	7101-	Oak Bay, WA	48.4	−123.3	1
53338	2400-	Puget Sound, WA	47.8	−122.4	12
53339	2890-	Seattle, WA	47.6	−122.3	73
53340	E1310	Davis Bay, Lopez Island, WA	48.5	−122.9	11
53341	E1534	Garrison Bay, San Juan Island, WA	48.6	−123.2	2
53342	R1687	San Juan Island, WA	48.5	−123.1	24
53343	2770-	San Juan Island, WA	48.5	−123.1	147
53344	2911-	Olympia, WA	47	−122.9	9
53346	2438-	Neah Bay, WA	48.4	−124.6	2
53347	R1689	Port Angeles, WA	48.1	−123.4	6
53348	E1281	Anacortes, WA	48.5	−122.6	1
53349	R1689	Port Angeles, WA	48.1	−123.4	27
53350	B358	Neah Bay, WA	48.4	−124.6	2
53351	B360	Near Orchard Point, WA	47.6	−122.5	5
53352	R3446	Olympia, WA	47	−122.9	3
53353	R7153	Agate Beach, WA	48.4	−122.9	4
53354	R3445	Waterman Point, WA	47.6	−122.6	2
53355	R1689	Port Angeles, WA	48.1	−123.4	56
53364	2910-	Port Harford, CA	35.2	−120.8	4
53369	IP14348	Intake Cove, Diablo Canyon Power Plant, CA	35.2	−120.9	1
53370	E747	Horseshoe Cove, CA	38.3	−123.1	1
53371	E6101	Mussel Point, CA	38.3	−123.1	23
53372	2412-	Bolinas Bay, CA	37.9	−122.7	2
53373	2410-	Half Moon Bay, CA	37.5	−122.4	9
53374	E6914	South side of Punta Banda, Baja	31.7	−116.7	1
53375	E6919	Shell Beach, La Jolla, CA	32.8	−117.3	1
53376	E660	One mile north of Pigeon Point, CA	37.2	−122.4	5
53377	D8919	Monterey, CA	36.6	−121.9	7
53378	A3991	Newport Beach, CA	33.6	−117.9	6
53379	E6916	East side of Point Dume, CA	34	−118.8	3
53380	E6917	Cape Yakataga, AK	60.1	−142.4	1
53381	B831	Ocean Beach, CA	32.7	−117.3	10
53382	2898-	Fort Point, CA	37.8	−122.5	4
53383	E6077	Shell Beach, Bodega Head, CA	38.4	−123.1	3
53384	D8915	Puget Sound, WA	47.8	−122.4	3
53385	E6906	Rosario Beach, WA	48.4	−122.7	12
53386	2390-	Santa Barbara, CA	34.4	−119.7	9
53387	2440-	Tobin Beach, CA	NA	NA	9
53388	R1693	San Simeon Point, CA	35.6	−121.2	13
53389	2780-	Santa Cruz Island, CA	34	−119.8	1
53390	E805	Fort Bragg Landing Beach, CA	39.4	−123.8	3
53391	E6915	San Simeon, CA	35.6	−121.2	3
53393	R1685	Golden Gate, CA	37.8	−122.5	12
53394	2402-	AK	NA	NA	15
53395	E6910	Laguna Beach, CA	33.5	−117.8	2
53396	E629	East Point, Santa Rosa Island, CA	33.9	−120	1
53397	E344	Drakes Estero, CA	38	−122.9	6
53398	E3168	Dana Point, CA	33.5	−117.7	1
53399	E249	One mile south of Hardy, CA	39.7	−123.8	12
53400	E1300	Minnesota Reef, San Juan Island, WA	48.5	−123	6
53401	R3445	Waterman Point, WA	47.6	−122.6	2
53404	D8920	Vancouver, BC	49.3	−123.1	1
53405	2408-	Laguna Bay, CA	35.3	−120.7	1
53406	E812	Point Arena, CA	38.9	−123.7	10
53407	E70	Carmet, CA	38.4	−123.1	4
53408	E6911	Asilomar, CA	36.6	−121.9	2
53409	E172	Duncans Point, CA	38.4	−123.1	8
53411	B835	Coronado, CA	32.7	−117.2	4
53412	D8920	Vancouver, BC	49.3	−123.1	4
53413	E6907	North side of Brown Island, WA	48.5	−123	13
53414	E2045	Frenchman's Reef, CA	37.5	−122.5	4
53415	2774-	Round Island, Baja	32.4	−117.2	2
53416	2403-	Santa Cruz, CA	37	−122	59
53417	2411-	Pigeon Point, CA	37.2	−122.4	6
53418	2776-	Santo Tomas, Baja	31.6	−116.4	1
53419	2439-	Bolinas Bay, CA	37.9	−122.7	1
53420	2892-	Tacoma, WA	47.3	−122.4	11
53421	2433-	San Nicolas Island, CA	33.2	−119.5	1
53422	2435-	Lagoon Head, Baja	NA	NA	2
53423	2881-	San Miguel Island, Santa Barbara, CA	34	−120.4	10
53424	E6080	Shell Beach, Sonoma, CA	38.4	−123.1	9
53425	E6908	Port Orford, OR	42.7	−124.5	9
53426	E290	Point Pinos, Pacific Grove, CA	36.6	−121.9	10
53427	E3838	Half mile north of Point Buchon, CA	35.3	−120.9	11
53428	D8920	Vancouver, BC	49.3	−123.1	12
53429	A4216	Bolinas Bay, CA	37.9	−122.7	5
53430	2395-	Monterey, CA	36.6	−121.9	77
53431	E874	Arena Cove, CA	38.9	−123.7	16
53432	2393-	San Francisco, CA	37.8	−122.4	9
53433	R1685	Golden Gate, CA	37.8	−122.5	55
53434	2412-	Bolinas Bay, CA	37.9	−122.7	3
53435	3117-	CA	NA	NA	14
53436	E820	North of Lighthouse Point, Arena, CA	39	−123.7	5
53437	E860	Stewart's Point, CA	38.7	−123.4	7
53438	2397-	Farallon Islands, CA	37.7	−123	4
53439	2391-	Santa Barbara Island, CA	33.5	−119	14
53440	E100	Moss Beach, Half Moon Bay, CA	37.5	−122.5	9
53441	2395-	Monterey, CA	36.6	−121.9	8
53443	IP443	Dana Point, CA	33.5	−117.7	6
53444	IP438	San Pedro, CA	33.7	−118.3	4
53445	E423	Del Mar Point, CA	38.7	−123.5	19
53446	E388	One mile north of San Simeon, CA	35.6	−121.2	8
53448	E1	Dillon Beach, CA	38.3	−123	4
53449	E98	Moss Beach, Half Moon Bay, CA	37.5	−122.5	9
53450	2412-	Bolinas Bay, CA	37.9	−122.7	1
53451	E154	Tomales Point, CA	38.2	−123	3
53452	E68	Carmet, CA	38.4	−123.1	1
53461	E260	Near Elk Creek, CA	39.1	−123.7	1
53462	2403-	Crescent Bay, WA	48.2	−123.7	59
53463	E6858	Asilomar, CA	36.6	−121.9	2
53464	E288	Point Pinos, Pacific Grove, CA	36.6	−121.9	1
53465	IP14571	Sunset Cliffs, Granger Street, San Diego, CA	32.7	−117.3	4
53466	L281	Trinidad to Magdalena Bay, Monetery, CA	NA	NA	5
53467	E226	Two miles south of Cape Mendocino, CA	40.4	−124.4	4
53468	2433-	San Nicolas Island, CA	33.2	−119.5	11
53469	3117-	Bolinas Bay, CA	37.9	−122.7	37
53470	2403-	Santa Cruz, CA	37	−122	7
53471	2395-	Monterey, CA	36.6	−121.9	1
53472	E480	Palos Verdes Point, Redondo Beach, CA	33.8	−118.4	8
53473	B876	Mission Beach, CA	32.8	−117.3	80
53474	2388-	San Pedro, CA	33.7	−118.3	8
53475	D8919	Monterey, CA	36.6	−121.9	3
53476	E6864	Laguna Tide Pools, Laguna Beach, CA	33.5	−117.8	1
53478	D7921	Los Angeles, CA	33.7	−118.3	6
53479	E6861	Venice, CA	34	−118.5	2
53480	E6863	Venice, CA	34	−118.5	6
53481	E536	Santa Rosa Island, Santa Barbara, CA	34	−120.1	1
53482	E688	Gaviota Beach, CA	34.5	−120.2	5
53483	IP450	Gaviota Beach, CA	34.5	−120.2	19
53484	3393-	Cayucos, CA	35.4	−120.9	15
53485	E1401	Monarch Bay, CA	33.5	−117.7	14
53486	2390-	Half Moon Bay OR Santa Barbara, CA	NA	NA	10
53487	E6859	Cabrillo Beach, CA	33.7	−118.3	7
53488	E2905	Halfway between Punta Mezquite and Punta Sal Si Puedes, Baja	32.1	−116.9	1
53489	E3920	Point Loma, CA	32.7	−117.2	3
53490	E2520	Solana Beach, Del Mar, CA	33	−117.3	6
53491	E2000	Point Loma, CA	32.7	−117.2	13
53492	2776-	Santo Tomas, Baja	31.6	−116.4	46
53493	2781-	Pacific Beach, CA	32.8	−117.3	45
53494	A3659	100 m south of San Diego, 20 m offshore, CA	32.7	−117.2	6
53495	2392-	San Diego, CA	32.7	−117.2	223
53496	E6862	Piedras Blancas Point, CA	35.7	−121.3	2
53497	A3991	Newport Beach, CA	33.6	−117.9	7
53498	E678	Whites Point, San Pedro Qd, CA	33.7	−118.3	23
53499	E480	Palos Verdes Point, Redondo Beach, CA	33.8	−118.4	1
53501	R7162	Twin Rivers, WA	48.2	−124	3
53502	E6865	Neah Bay, WA	48.4	−124.6	1
53503	3122-	OR	NA	NA	2
53504	IP441	Monterey, CA	36.6	−121.9	2
53505	E889	Arena Cove, CA	38.9	−123.7	12
53506	E424	Del Mar Point, CA	38.7	−123.5	6
53507	B361	Clallam Bay, WA	48.3	−124.3	3
53508	E3777	Stillwater Cove, CA	38.5	−123.3	10
53509	R7209	Pillar Point near Pysht, WA	48.2	−124.1	3
53510	E6351	Gualala, CA	38.8	−123.5	12
53511	D8919	Monterey, CA	36.6	−121.9	1
53512	E647	Pillar Point, Half Moon Bay, CA	37.5	−122.5	1
53513	L282	Neah Bay, WA	48.4	−124.6	2
53514	R7163	Port Crescent, WA	48.2	−123.7	6
53515	E765	Russian Gulch, CA	39.3	−123.8	1
53516	E189	Havens Neck, CA	38.8	−123.6	17
53517	R7173	Clallam Bay, WA	48.3	−124.3	7
53518	2438-	Neah Bay, WA	48.4	−124.6	8
53519	R7210	Freshwater Bay, WA	48.1	−123.6	3
53520	R7162	Twin Rivers, WA	48.2	−124	1
53521	E6205	Carmel Cove, CA	36.5	−121.9	6
53522	2420-	Crescent City, CA	41.8	−124.2	2
53523	2438-	Neah Bay, WA	48.4	−124.6	1
53524	E735	Shell Beach, Bodega Head, CA	38.4	−123.1	1
53525	E6867	North Point Conception, Santa Barbara, CA	34.4	−120.5	1
53526	B361	Clallam Bay, WA	48.3	−124.3	1
53527	E9961	Crescent City, CA	41.8	−124.2	14
53528	R7136	Northern CA	NA	NA	2
53530	R2438	WA	NA	NA	1
53531	E719	Salt Point, CA	38.6	−123.3	1
53532	E6866	Granite Creek Canyon off Carmel, CA	36.5	−122	4
53534	2411-	Pigeon Point, CA	37.2	−122.4	4
53535	2392-	San Diego, CA	32.7	−117.2	2
53536	2392-	San Diego Jetty, San Diego, CA	32.7	−117.2	7
53537	E6900	Asilomar, CA	36.6	−121.9	2
53538	IP14539	Punta Abreojos, Baja	26.7	−113.6	22
53539	E587	Southeast corner of San Nicolas Island, CA	33.2	−119.5	4
53540	3117-	Moreno, CA	NA	NA	5
53541	2392-	San Diego, CA	32.7	−117.2	6
53542	E3647	Estero de Punta Banda, Baja	31.7	−116.7	10
53543	2388-	San Pedro, CA	33.7	−118.3	2
53544	B829	Mission Bay, CA	32.8	−117.2	6
53545	2409-	La Jolla, CA	32.8	−117.3	2
53546	E2540	Flood Control Channel, San Diego, CA	32.8	−117.3	1
53547	D8930	San Diego, CA	32.7	−117.2	4
53548	IP14593	Flood Control Channel, San Diego, CA	32.8	−117.3	1
53549	D9894	Estero de Punta Banda, Baja	31.7	−116.7	1
53550	2390-	Santa Barbara, CA	34.4	−119.7	8
53551	IP449	Willows Cove, Santa Cruz Island, CA	34	−119.8	1
53552	IP10591	Tatoosh Island, WA	48.4	−124.7	6
53553	E6079	Shell Beach, Sonoma, CA	38.4	−123.1	1
53554	E574	South Anacapa Island, Ventura, CA	34	−119.4	12
53555	E6078	Shell Beach, Bodega Head, CA	38.4	−123.1	2
53557	E4447	Puertecitos, Baja	30.3	−114.6	4
53560	E4504	Just north of Puertecitos, Baja	30.4	−114.6	13
53562	E4556	Five miles south of Puertecitos, Baja	30.3	−114.7	5
53564	E6966	One mile south of Puertecitos, Baja	30.3	−114.6	5
53565	IP446	Gaviota Beach, CA	34.5	−120.2	7
53569	2404-	Gulf of CA	30.3	−113.7	1
53571	E4369	San Luis Gonzaga Bay, Baja	29.8	−114.4	8
53572	E4511	Twenty-eight miles south of Puertecitos, Baja	30	−114.5	29
53574	E4555	Five miles south of Puertecitos, Baja	30.3	−114.7	4
53575	E6966	Puertecitos, Baja	30.3	−114.6	1
53577	E4576	Nineteen miles south of Puertecitos, Baja	30.1	−114.6	1
53578	IP442	San Felipe, Baja	31	−114.8	12
53582	E4372	San Luis Gonzaga Bay, Baja	29.8	−114.4	2
53583	E5228	Puertecitos, Baja	30.3	−114.6	5
53584	E4224	Two miles north of Puertecitos, Baja	30.4	−114.6	3
53586	E2426	Los Angeles Bay, Baja	28.9	−113.6	3
53588	E4575	Nineteen miles south of Puertecitos, Baja	30.1	−114.6	15
53589	E4842	Five miles south of Puertecitos, Baja	30.3	−114.7	3
53591	2404-	Gulf of CA	30.3	−113.7	10
53593	E4371	San Luis Gonzaga Bay, Baja	29.8	−114.4	17
53594	6119-	La Paz, Baja	24.1	−110.3	2
53598	IP442	San Felipe, Baja	31	−114.8	1
53605	E6967	Puertecitos, Baja	30.3	−114.6	1
53606	E6959	Puertecitos, Baja	30.3	−114.6	3
53611	6119-	La Paz, Baja	24.1	−110.3	16
53615	E3007	San Felipe, Baja	31	−114.8	29
53620	E4864	Paralof Bay, AK	57.9	−135.8	4
53625	IP14654	Bird Rock, CA	32.8	−117.3	1
53627	E5346	Palos Verdes Point, Redondo Beach, CA	33.8	−118.4	2
53628	E5346	Palos Verdes Point, Redondo Beach, CA	33.8	−118.4	3
53632	IP451	Dana Point, CA	33.5	−117.7	6
53633	IP452	Dana Point, CA	33.5	−117.7	5
53634	IP453	Dana Point, CA	33.5	−117.7	4
53637	E3061	Punta Banda, Baja	31.7	−116.7	12
53638	B829	Mission Bay, CA	32.8	−117.2	26
53639	E4026	San Quintin Bay, Baja	30.6	−115.9	7
53641	E3910	Rosarita Beach, Baja	32.3	−117.1	37
53643	IP14596	Sunset Cliffs, Hill Street, San Diego, CA	32.7	−117.3	3
53644	E1963	South of Rio Guadalupe, Baja	32.1	−116.5	16
53645	E452	Dana Point, CA	33.5	−117.7	16
53646	IP14593	San Diego, CA	32.7	−117.2	2
53647	E577	Johnson's Landing, Santa Catalina Island, CA	33.5	−118.5	13
53648	E1973	Halfway Reef, Baja	0	0	8
53649	E491	Rincon Beach, CA	34.3	−119.4	18
53650	E2541	Flood Control Channel, San Diego, CA	32.8	−117.3	3
53651	E3252	South of La Mision River, Baja	32.1	−116.9	16
53652	2436-	Golden Gate, CA	37.8	−122.5	16
53654	R1688	San Diego Jetty, San Diego, CA	32.7	−117.2	2
53655	E6897	Shell Beach, Bodega Head, CA	38.4	−123.1	4
53656	E6896	Monterey, CA	36.6	−121.9	4
53659	E6899	Arch Rock, CA	38	−122.8	7
53660	E570	North Anacapa Island, CA	34	−119.4	1
53661	2392-	San Diego, CA	32.7	−117.2	1
53662	2392-	San Diego, CA	32.7	−117.2	15
53665	E6898	Timber Cove, CA	38.5	−123.3	3
53667	E1999	Point Loma, CA	32.7	−117.2	11
53668	E9989	Flood Control Channel, San Diego, CA	32.8	−117.3	11
53669	IP14355	Punta Baja, Baja	29.9	−115.8	3
53670	IP14358	Nobi Point, Port San Luis, CA	35.2	−120.8	1
53671	IP14352	North Diablo Cove, San Luis Obispo, CA	35.2	−120.9	1
53672	IP14361	South Diablo Cove, San Luis Obispo, CA	35.2	−120.9	1
53675	IP440	La Jolla, CA	32.8	−117.3	2
53676	E3909	Five miles north of Rosarita Beach, Baja	32.4	−117.1	1
53678	IP14357	Scorpion Cove, Santa Cruz Island, CA	34	−119.8	1
53679	E1973	Halfway Reef, Baja	0	0	1
53680	E1957	South of Punta Abreojos, Baja	26.8	−113.5	7
53681	E1973	Halfway Reef, Baja	0	0	8
53682	IP14350	South Diablo Cove, San Luis Obispo, CA	35.2	−120.9	1
130038	E6350	Newport Bay, CA	33.6	−117.9	3
130100	2395-	Monterey, CA	36.6	−121.9	44
130101	2388-	San Pedro, CA	33.7	−118.3	74
130102	A4198	Ensenada, Baja	31.9	−116.6	38
130103	E1385	South of Scripps, La Jolla, CA	32.9	−117.3	35
130104	E699	Morro Rock, CA	35.4	−120.9	1
130105	A4198	Ensenada, Baja	31.9	−116.6	36
130106	E3490	Punta Final, Baja	29.8	−114.3	35
130108	E3664	Estero de Punta Banda, Baja	31.7	−116.7	5
130109	E616	San Miguel Island, Santa Barbara, CA	34	−120.4	14
130110	E1919	Southeast Coronado Island, Los Coronados, Baja	32.4	−117.2	74
130111	E1907	Southeast Coronado Island, Los Coronados, Baja	32.4	−117.2	30
130112	2395-	Monterey, CA	36.6	−121.9	5
130113	E756	Santa Cruz, CA	37	−122	2
130114	E529	Willows Anchorage, Santa Cruz Island, CA	34	−119.8	24
130115	E6205	Carmel Cove, CA	36.5	−121.9	6
130116	2395-	Monterey, CA	36.6	−121.9	16
130117	A4232	Davenport, CA	37	−122.2	1
130118	D9226A	Monterey, CA	36.6	−121.9	2
130119	2390-	Santa Barbara, CA	34.4	−119.7	2
130120	D9340	Southeast Farallon Island, CA	37.7	−123	1
130121	R516	Bear Bay, Baranoff Island, AK	57.5	−135.5	3
130122	2404-	Gulf of CA	30.3	−113.7	3
130123	E5310	Estero de Bahia de Todas Santos, Baja	27.7	−114.9	9
130124	2390-	Santa Barbara, CA	34.4	−119.7	1
130125	E6526	Cabo San Lucas, Baja	22.9	−109.9	8
130126	E3434	San Felipe, Baja	31	−114.8	19
130127	IP10589	Tatoosh Island, WA	48.4	−124.7	1
130128	2392-	San Diego, CA	32.7	−117.2	14
130129	2392-	San Diego, CA	32.7	−117.2	12
130131	R1695	Tomales Bay, CA	38.2	−122.9	3
130132	R1680	Santa Cruz, CA	37	−122	40
130133	R1694	Bolinas Bay, CA	37.9	−122.7	9
130134	R1680	Santa Cruz Island, CA	34	−119.8	39
130135	2770-	San Juan Island, WA	48.5	−123.1	14
130136	R1694	Bolinas Bay, CA	37.9	−122.7	3
130137	R1678	Seattle, WA	47.6	−122.3	20
130138	R1698	Monterey, CA	36.6	−121.9	24
130139	R1677	Port Crescent, WA	48.2	−123.7	5
130140	R1753	Pacific Grove, CA	36.6	−121.9	2
130141	R1752	Pacific Grove, CA	36.6	−121.9	8
130142	2439-	Bolinas Bay, CA	37.9	−122.7	24
130143	R1694	Bolinas Bay, CA	37.9	−122.7	12
130145	R1693	San Simeon Point, CA	35.6	−121.2	15
130146	R1668	San Diego, CA	32.7	−117.2	22
130147	2770-	San Juan Island, WA	48.5	−123.1	32
130148	R1668	San Diego Jetty, San Diego, CA	32.7	−117.2	34
130149	2770-	San Juan Island, WA	48.5	−123.1	15
130150	R1678	Seattle, WA	47.6	−122.3	16
130151	2786-	San Simeon Point, CA	35.6	−121.2	46
130152	R1676	Neah Bay, WA	48.4	−124.6	10
130153	2787-	Fort Bragg, CA	39.4	−123.8	49
130154	R1694	Bolinas Bay, CA	37.9	−122.7	22
130155	2392-	San Diego, CA	32.7	−117.2	121
130156	2786-	San Simeon Point, CA	35.6	−121.2	35
130157	R1752	Pacific Grove, CA	36.6	−121.9	1
130158	R1668	San Diego, CA	32.7	−117.2	47
130163	R1695	Tomales Bay, CA	38.2	−122.9	9
130166	R1693	San Simeon Point, CA	35.6	−121.2	5
130168	R1693	San Simeon Point, CA	35.6	−121.2	5
130169	R1694	Bolinas Bay, CA	37.9	−122.7	3
130170	R1671	Bahia Tortugas, Baja	27.7	−114.9	21
130171	R1695	Tomales Bay, CA	38.2	−122.9	64
130172	R1690	Santa Barbara Island, CA	33.5	−119	49
130173	R1690	Santa Barbara Island, CA	33.5	−119	2
130174	R1690	Santa Barbara Island, CA	33.5	−119	2
130175	R1693	San Simeon Point, CA	35.6	−121.2	11
130176	R1693	San Simeon Point, CA	35.6	−121.2	1
130177	R1693	San Simeon Point, CA	35.6	−121.2	4
130178	1753	Monterey, CA	36.6	−121.9	2
130179	R1754	Tomales Bay, CA	38.2	−122.9	1
130180	R1685	Golden Gate, CA	37.8	−122.5	5
130181	R1685	Golden Gate, CA	37.8	−122.5	26
130183	R1752	Pacific Grove, CA	36.6	−121.9	1
130184	R1752	Pacific Grove, CA	36.6	−121.9	1
130185	E508	Moss Landing, CA	36.8	−121.8	1
130186	E1957	South of Punta Abreojos, Baja	26.8	−113.5	19
130187	E1957	South of Punta Abreojos, Baja	26.8	−113.5	3
130188	E1973	Halfway Reef, Baja	0	0	7
130189	E3707	Mission Bay Breakwater, San Diego, CA	32.8	−117.2	4
130190	E1919	South Coronado Island, Baja	32.4	−117.2	1
130191	E1919	South Coronado Island, Baja	32.4	−117.2	1
130192	E6901	Venice, CA	34	−118.5	49
130193	E2519	Solana Beach, Del Mar, CA	33	−117.3	2
130194	E3115	Punta Piedra, Baja	32.1	−116.9	12
130195	E4979	Estero de Punta Banda, Baja	31.7	−116.7	16
130196	E1973	Halfway Reef, Baja	0	0	5
130198	2395-	Monterey, CA	36.6	−121.9	8
130199	R7162	Twin Rivers, WA	48.2	−124	20
130200	R4093	Beaver Cove, Vancouver Island, BC	50.5	−126.9	1
130201	E887	Arena Cove, CA	38.9	−123.7	12
130202	E184	Havens Neck, CA	38.8	−123.6	23
130203	R517	Red Bluff Bay, Baranoff Island, AK	56.9	−134.8	3
130204	E6503	Mendocino, CA	39.3	−123.8	11
130205	E892	Pigeon Point, CA	37.2	−122.4	5
130206	2412-	Bolinas Bay, CA	37.9	−122.7	7
130207	2423-	Purissima, CA	37.4	−122.4	2
130208	2393-	San Francisco, CA	37.8	−122.4	7
130209	R5046	Hunters Point, CA	37.7	−122.4	1
130210	2436-	Golden Gate, CA	37.8	−122.5	3
130211	2392-	San Diego, CA	32.7	−117.2	3
130212	R1685	Golden Gate, CA	37.8	−122.5	2
130213	2787-	Fort Bragg, CA	39.4	−123.8	1
130214	2411-	Pigeon Point, CA	37.2	−122.4	13
130215	E734	Shell Beach, Bodega Head, CA	38.4	−123.1	4
130216	E171	Duncans Point, CA	38.4	−123.1	5
130217	E2121	Bodega Head, CA	38.3	−123.1	2
130218	E416	Duxbury Point, CA	37.9	−122.7	3
130219	E718	Salt Point, CA	38.6	−123.3	3
130220	E422	Del Mar Point, CA	38.7	−123.5	1
130221	2890-	Seattle, WA	47.6	−122.3	1
130222	3094-	Mendocino, CA	39.3	−123.8	3
130223	3342-	West Vancouver Island, BC	49.5	−126.6	2
130224	R7210	Freshwater Bay, WA	48.1	−123.6	1
130225	2439-	Bolinas, CA	37.9	−122.7	55
130226	E903	Carmet, CA	38.4	−123.1	3
130227	E215	North of Redwood Creek, Orick, CA	41.3	−124.1	7
130228	E519	Two miles south of San Simeon, CA	35.6	−121.2	4
130229	E901	Richmond Point, CA	37.9	−122.4	1
130230	E902	Dillon Beach, CA	38.3	−123	2
130231	E343	Drakes Estero, CA	38	−122.9	2
130232	E245	One mile south of Hardy, CA	39.7	−123.8	11
130233	E1962	South of Rio Guadalupe, Baja	32.1	−116.5	3
130234	E666	Near mouth of Scott Creek, Davenport, CA	37	−122.2	9
130235	E178	Russian Gulch, CA	39.3	−123.8	6
130236	E804	Fort Bragg Landing Beach, CA	39.4	−123.8	1
130237	IP10590	Tatoosh Island, WA	48.4	−124.7	1
130238	E1261	Del Mar Ranch, CA	38.7	−123.5	32
130239	3094-	Mendocino, CA	39.3	−123.8	4
130240	E596	Isthmus Cove, Santa Catalina Island, CA	33.4	−118.5	4
130241	E510	Moonstone Beach, Cambria, CA	35.6	−121.1	12
130242	E6856	East side of Point Dume, CA	34	−118.8	3
130243	E1973	Halfway Reef, Baja	0	0	2
130244	E1973	Halfway Reef, Baja	0	0	16
130245	E198	Near CA-OR border, Smith River, CA	41.9	−124.2	11
130246	E6854	Moss Beach, Half Moon Bay, CA	37.5	−122.5	6
130247	E38	Duxbury Reef, CA	37.9	−122.7	1
130248	E3904	Five miles north of Rosarita Beach, Baja	32.4	−117.1	1
130249	A4216	Bolinas Bay, CA	37.9	−122.7	4
130250	A4217	Stinson Beach, CA	37.9	−122.6	9
130251	E612	South end of Cuyler Harbor, San Miguel Island, CA	34	−120.4	2
130252	E638	Rincon Beach, CA	34.3	−119.4	1
130253	IP14364	Intake Cove, Diablo Canyon Power Plant, CA	35.2	−120.9	1
130254	E446	Three miles east of Gaviota, CA	34.5	−120.2	14
130255	E446	Three miles east of Gaviota, CA	34.5	−120.2	5
130256	E687	Gaviota Beach, CA	34.5	−120.2	17
130257	E224	Two miles south of Cape Mendocino, CA	40.4	−124.4	2
130258	E643	Carpinteria, CA	34.4	−119.5	9
130259	E2790	Half mile north of Dana Point, CA	33.5	−117.7	4
130260	E376	Government Point, CA	34.4	−120.5	26
130261	2436-	Golden Gate, CA	37.8	−122.5	32
130262	R3467	Rocky Point Dye Inlet, Bremerton, WA	47.6	−122.7	6
130263	D8919	Tiburon, CA	37.9	−122.5	23
130264	E821	Point Arena, CA	38.9	−123.7	6
130265	E6920	Port Orford, OR	42.7	−124.5	6
130266	E186	Havens Neck, CA	38.8	−123.6	7
130267	E6391	Red Bluff Bay, Baranoff Island, AK	56.9	−134.8	9
130268	E180	Russian Gulch, CA	39.3	−123.8	7
130269	D8930	San Diego, CA	32.7	−117.2	3
130270	E9990	Puget Sound, WA	47.8	−122.4	1
130271	E738	Shell Beach, Bodega Head, CA	38.4	−123.1	8
130272	E216	North of Redwood Creek, Orick, CA	41.3	−124.1	1
130273	E6920	Neah Bay, WA	48.4	−124.6	2
130274	E250	One mile south of Hardy, CA	39.7	−123.8	2
130275	E345	Drakes Estero, CA	38	−122.9	5
130276	B7062	Green Cove, Admiralty Island, AK	58.2	−134.3	8
130277	E1301	San Juan Island, WA	48.5	−123.1	5
130278	E262	Near mouth of Elk Creek, South of Elk Creek, CA	39.1	−123.7	13
130279	E6924	Arch Rock, CA	38	−122.8	4
130280	2414-	Waldron Island, WA	48.7	−123	3
130281	E4608	San Luis Gonzaga Bay, Baja	29.8	−114.4	2
130282	IP16028	Middle Coronados Island, Baja	32.4	−117.3	1
130284	E2883	Off mouth of Tijuana River, Imperial Beach, CA	32.6	-117.1	1
130285	E2267	San Diego, CA	32.7	-117.2	9
130286	E2476	San Diego, CA	32.7	-117.2	9
130287	2392-	San Diego, CA	32.7	-117.2	4
130288	2395-	Monterey, CA	36.6	-121.9	1
130293	E619	San Miguel Island, Santa Barbara, CA	34	-120.4	12
130294	E3832	West of Canada Tecelate, Santa Rosa Island, CA	34	-120.1	2
130296	E1969	South of Rio Guadalupe, Baja	32.1	-116.5	1
130299	E692	Gaviota Beach, CA	34.5	-120.2	17
130300	E1391	Two miles south of Scripps, La Jolla, CA	32.8	-117.3	7
130301	IP14708	Little Corona Beach, Corona del Mar, CA	33.6	-117.9	1
130302	E1464	North of Scripps, La Jolla, CA	32.9	-117.3	8
130303	2408-	Laguna Bay, CA	35.3	-120.7	14
130304	D290	The Nursery, Isla Guadalupe, Baja	29	-118.3	1
130305	D1915	Isla Guadalupe, Baja	29	-118.3	2
130306	E9936	San Diego, CA	32.7	-117.2	2
130307	E1411	Half mile north of Dana Point, CA	33.5	-117.7	4
130308	E3191	Dana Point, CA	33.5	-117.7	1
130309	A9119	Punta Santo Tomas, Baja	31.6	-116.7	1
130310	D289	Barracks Beach, Baja	29	-118.3	1
130311	E3258	South of La Mision River, Baja	32.1	-116.9	6
130312	B1412	South side of Punta Banda, Baja	31.7	-116.7	1
130313	B3057	Bahia Tortugas, Baja	27.7	-114.9	5
130316	B1412	South side of Punta Banda, Baja	31.7	-116.7	2
130317	E3069	Punta Banda, Baja	31.7	-116.7	1
130318	E3915	Five miles north of Rosarita Beach, Baja	32.4	-117.1	4
130321	E1987	Halfway between Punta Mezquite and Punta Sal Si Puedes, Baja	32.1	-116.9	2
130322	E3120	Punta Piedra, Baja	32.1	-116.9	1
130323	2419-	Moss Beach, Half Moon Bay, CA	37.5	-122.5	9
130324	R520	Mole Harbor, Admiralty Island, AK	57.7	-134.1	3
130325	2786-	San Simeon Point, CA	35.6	-121.2	1
130326	2440-	Tobin Beach, CA	0	0	45
130327	2388-	San Pedro, CA	33.7	-118.3	4
130328	2428-	Coos Bay, OR	43.4	-124.2	3
130329	R519	Windfall Harbor, Admiralty Island, AK	57.9	-134.3	8
130330	R7162	Twin Rivers, WA	48.2	-124	8
130331	2787-	Fort Bragg, CA	39.4	-123.8	94
130332	2435-	Lagoon Head, Baja	NA	NA	4
130333	2896-	Sitka, AK	57.1	-135.3	7
130334	R7149	Little River, CA	39.3	-123.8	1
130335	3341-	Kirby Creek, Vancouver Island, BC	48.4	-123.9	1
130336	R3400	Black Point, CA	38.7	-123.4	2
130337	R7163	Port Crescent, WA	48.2	-123.7	1
130338	2440-	Tobin Beach, CA	0	0	3
130339	A4216	Bolinas Bay, CA	37.9	-122.7	3
130340	2393-	San Francisco, CA	37.8	-122.4	11
130341	2395-	Monterey, CA	36.6	-121.9	1
130342	R4093	Beaver Cove, Vancouver Island, BC	50.5	-126.9	1
130343	R513	Anacortes, WA	48.5	-122.6	1
130344	3394-	Victoria, BC	48.4	-123.4	3
130345	R7174	Restoration Point, Bainbridge Island, WA	47.6	-122.5	5
130346	R518	Glacier Bay, AK	58.8	-136.3	1
130347	R526	Hawk Inlet, AK	58.1	-134.8	2
130348	2436-	Golden Gate, CA	37.8	-122.5	7
130349	R1637	East coast of Graham Island, BC	53.4	-131.9	2
130350	2435-	Lagoon Head, Baja	NA	NA	6
130351	B359	Neah Bay, WA	48.4	-124.6	2
130352	E6461	Cape Mendocino, CA	40.4	-124.4	8
130353	R525	Head of Port Frederick, AK	58.1	-135.6	2
130354	R1677	Port Crescent, WA	48.2	-123.7	25
130355	B359	Waddah Island, Neah Bay, WA	48.4	-124.6	8
130356	B6645-	Annette Island, AK	55.1	-131.4	1
130357	B6644-	Annette Island, AK	55.1	-131.4	3
130358	2439-	Bolinas, CA	37.9	-122.7	2
130359	2439-	Bolinas, CA	37.9	-122.7	1
130360	2432-	Port Townsend, WA	48.1	-122.8	5
130361	2432-	Port Townsend, WA	48.1	-122.8	1
130362	B1260	Pacific Grove, CA	36.6	-121.9	2
130363	R5033	Popof Island, AK	55.3	-160.4	2
130364	B7061	Point Louisa, AK	58.4	-134.7	7
130365	2395-	Monterey, CA	36.6	-121.9	18
130366	IP14363	North of Pier, Cayucos Beach, CA	35.4	-120.9	1
130367	E6075	Shell Beach, Bodega Head, CA	38.4	-123.1	14
130368	D8919	Moss Beach, Half Moon Bay, CA	37.5	-122.5	1
130369	D8919	Moss Beach, Half Moon Bay, CA	37.5	-122.5	35
130370	2395-	Monterey, CA	36.6	-121.9	37
130371	E875	Point Arena, CA	38.9	-123.7	16
130372	E1280	Anacortes, WA	48.5	-122.6	8
130373	E1288	False Bay, San Juan Island, WA	48.5	-123.1	2
130374	E1329	Brown Island, Friday Harbor, WA	48.5	-123	11
130375	E2123	Bodega Head, CA	38.3	-123.1	5
130376	E2232	Bodega Harbor, CA	38.3	-123	7
130377	E4864	Pavalof Bay, Chichagof Island, AK	55.5	-161.5	9
130378	E6076	Shell Beach, Bodega Head, CA	38.4	-123.1	4
130379	E6510	Point Loma Lighthouse, CA	32.7	-117.2	2
130381	E6923	Point Chehalis, WA	46.9	-124.1	2
130382	2787-	Fort Bragg, CA	39.4	-123.8	61
130383	E229	Two miles south of Cape Mendocino, CA	40.4	-124.4	6
130384	E405	Brown Island, Friday Harbor, WA	48.5	-123	36
130385	E429	Del Mar Point, CA	38.7	-123.5	1
130386	E723	Salt Point, CA	38.6	-123.3	4
130387	E771	Yakutat, AK	59.5	-139.7	20
130388	E774	Mussel Point, CA	38.3	-123.1	7
130389	E806	Fort Bragg Landing Beach, CA	39.4	-123.8	15
130390	E71	Carmet, CA	38.4	-123.1	5
130391	R3400	Black Point, CA	55.4	-161.7	3
130392	R7162	Twin Rivers, WA	48.2	-124	16
130393	A8737	Yakutat, AK	59.5	-139.7	25
130394	R7209	Pillar Point near Pysht, WA	48.2	-124.1	1
130395	A4198	Ensenada, Baja	31.9	-116.6	4
130396	A4218	Dillon Beach, CA	38.3	-123	5
130397	B358	Neah Bay, WA	48.4	-124.6	2
130398	B6644-	Annette Island, AK	55.1	-131.4	3
130399	E33	Richmond Point, CA	37.9	-122.4	1
130400	E52	Dillon Beach, CA	38.3	-123	2
130401	E200	Near CA-OR border, Smith River, CA	41.9	-124.2	4
130402	3117-	CA	NA	NA	47
130403	E193	North end of Klamath Cove, Requia, CA	41.5	-124.1	5
130404	3122-	Tillamook, OR	45.5	-123.8	20
130405	3341-	Between Muir and Kirby Creek, Vancouver Island, BC	48.4	-123.9	5
130406	3342-	West Vancouver Island, BC	49.5	-126.6	4
130407	R4093	Beaver Cove, Vancouver Island, BC	50.5	-126.9	1
130408	R5046	Hunters Point, CA	37.7	-122.4	4
130409	E196	South end of False Klamath Cove, Requia, CA	41.6	-124.1	7
130410	D8919	Tiburon, CA	37.9	-122.5	44
130411	2780-	Santa Cruz Island, CA	34	-119.8	13
130412	2785-	Russian Gulch, CA	39.3	-123.8	12
130413	2787-	Fort Bragg, CA	39.4	-123.8	24
130414	2898-	Fort Point, CA	37.8	-122.5	7
130415	2888-	Port Crescent, WA	48.2	-123.7	4
130416	2890-	Seattle, WA	47.6	-122.3	6
130417	2909-	Hood Canal, WA	47.6	-123	5
130418	3101-	Vancouver Island, BC	49.7	-125.8	3
130420	2432-	Port Townsend, WA	48.1	-122.8	13
130421	2434-	Bahia Tortugas, Baja	27.7	-114.9	35
130422	2434-	Bahia Tortugas, Baja	27.7	-114.9	39
130423	2435-	Lagoon Head, Baja	NA	NA	3
130424	2436-	Golden Gate, CA	37.8	-122.5	62
130425	2438-	Neah Bay, WA	48.4	-124.6	4
130426	2439-	Bolinas, CA	37.9	-122.7	13
130427	2439-	Bolinas, CA	37.9	-122.7	43
130428	2439-	Bolinas, CA	37.9	-122.7	32
130429	2496-	Humboldt County, CA	40.7	-124.2	1
130430	2770-	San Juan Island, WA	48.5	-123.1	32
130431	2773-	Tomales Bay, CA	38.2	-122.9	9
130432	2423-	Purissima, CA	37.4	-122.4	2
130433	2428-	Coos Bay, OR	43.4	-124.2	4
130434	2393-	San Francisco, CA	37.8	-122.4	4
130435	2394-	South of Mission, San Francisco, CA	37.7	-122.4	2
130438	2395-	Monterey, CA	36.6	-121.9	1
130439	2400-	Puget Sound, WA	47.8	-122.4	11
130440	2403-	Santa Cruz, CA	37	-122	10
130441	2405-	Sucia Islands, WA	48.8	-122.9	19
130442	2409-	La Jolla, CA	32.8	-117.3	5
130443	2411-	Pigeon Point, CA	37.2	-122.4	7
130444	R516	Bear Bay, Baranoff Island, AK	57.5	-135.5	5
130445	R518	Glacier Bay, AK	58.8	-136.3	5
130446	2388-	San Pedro, CA	33.7	-118.3	10
130447	2390-	Santa Barbara, CA	34.4	-119.7	6
130448	2890-	Bolinas Bay, CA	37.9	-122.7	1
130449	2390-	Santa Barbara, CA	34.4	-119.7	111
130450	2391-	Santa Barbara Island, CA	33.5	-119	16
130451	2392-	San Diego, CA	32.7	-117.2	50
130452	2392-	San Diego, CA	32.7	-117.2	122
130454	E873	Frazer Point, Santa Cruz Island, CA	34	-119.8	5
130458	E2590	Bird Rock, CA	32.8	-117.3	1
130459	2485-	Pescadero, CA	37.3	-122.4	1
130460	IP14591	Sunset Cliffs, San Diego, CA	32.7	-117.3	11
130461	A3991	Newport Beach, CA	33.6	-117.9	2
130463	E6833	Lunada Bay, CA	33.8	-118.4	5
130464	B841	Bird Rock, CA	32.8	-117.3	2
130465	B835	Coronado, CA	32.7	-117.2	1
130466	IP14596	Sunset Cliffs, Hill Street, San Diego, CA	32.7	-117.3	1
130467	IP14597	Point Loma Lighthouse, CA	32.7	-117.2	1
130468	2409-	La Jolla, CA	32.8	-117.3	1
130469	E363	Horseshoe Point, CA	38.6	-123.4	1
130471	E566	Moonstone Beach, Santa Catalina Island, CA	33.4	-118.4	1
130474	2395-	Monterey, CA	36.6	-121.9	11
130475	2395-	Monterey, CA	36.6	-121.9	11
130477	E790	Soberanes Point, CA	36.4	-121.9	3
130479	E692	Santa Barbara, CA	34.4	-119.7	2
130480	E3841	Half mile north of Point Buchon, CA	35.3	-120.9	2
130481	D8930	San Diego, CA	32.7	-117.2	1
130482	E382	Government Point, CA	34.4	-120.5	9
130483	D8919	Monterey, CA	36.6	-121.9	3
130484	2410-	Half Moon Bay, CA	37.5	-122.4	3
130488	R5035	Pacific Grove, CA	36.6	-121.9	1
130489	R1775	Point Pinos, Pacific Grove, CA	36.6	-121.9	2
130490	2419-	Moss Beach, Half Moon Bay, CA	37.5	-122.5	2
130491	E584	Santa Barbara Island, CA	33.5	-119	5
130492	E1248	Pigeon Point, CA	37.2	-122.4	2
130495	2390-	Santa Barbara, CA	34.4	-119.7	8
130496	E592	Southeast corner of San Nicolas Island, CA	33.2	-119.5	1
130498	2411-	Pigeon Point, CA	37.2	-122.4	2
130499	E6834	Arch Rock, CA	38	-122.8	1
130500	E639	Rincon Beach, CA	34.3	-119.4	11
130501	2411-	Pigeon Point, CA	37.2	-122.4	6
130502	E548	Santa Rosa Island, Santa Barbara, CA	34	-120.1	2
130503	E920	Tomales Point, CA	38.2	-123	1
130504	2395-	Monterey, CA	36.6	-121.9	3
130505	2395-	Monterey, CA	36.6	-121.9	43
130506	2395-	Monterey, CA	36.6	-121.9	2
130507	E3904	Five miles north of Rosarita Beach, Baja	32.4	-117.1	1
130508	E1149	One mile north of Pigeon Point, CA	37.2	-122.4	2
130509	2395-	Monterey, CA	36.6	-121.9	11
130510	E807	Fort Bragg Landing Beach, CA	39.4	-123.8	1
130511	2395-	Monterey, CA	36.6	-121.9	2
130512	D8919	Mussel Point, CA	38.3	-123.1	1
130513	D8919	Tiburon, CA	37.9	-122.5	13
130514	E9989	Flood Control Channel, San Diego, CA	32.8	-117.3	7
130515	2394-	San Francisco, CA	37.8	-122.4	3
130516	2394-	San Francisco, CA	37.8	-122.4	12
130517	E571	North Anacapa Island, CA	34	-119.4	3
130518	2771-	Coronado Island, CA	32.7	-117.2	5
130519	2771-	Coronado Island, CA	32.7	-117.2	3
130520	2771-	Coronado Island, CA	32.7	-117.2	1
130521	E360	Horseshoe Point, CA	38.6	-123.4	3
130522	E520	Two miles south of San Simeon, CA	35.6	-121.2	9
130523	E6929	Timber Cove, CA	38.5	-123.3	23
130524	E6929	Timber Cove, CA	38.5	-123.3	3
130525	E681	Whites Point, San Pedro Qd, CA	33.7	-118.3	6
130526	D8930	San Diego, CA	32.7	-117.2	4
130527	E444	Dana Point, CA	33.5	-117.7	10
130528	D8919	Moss Beach, Half Moon Bay, CA	37.5	-122.5	5
130529	D8919	Monterey, CA	36.6	-121.9	3
130530	2388-	San Pedro, CA	33.7	-118.3	4
130531	E498	Rincon Beach, CA	34.3	-119.4	5
130532	E775	Mussel Point, CA	38.3	-123.1	2
130533	E775	Mussel Point, CA	38.3	-123.1	1
130534	E775	Mussel Point, CA	38.3	-123.1	1
130535	E2233	Bodega Harbor, CA	38.3	-123	9
130536	E6496	North Jetty, Humboldt Bay, CA	40.7	-124.2	12
130537	E6927	Asilomar, CA	36.6	-121.9	1
130538	E379	Government Point, CA	34.4	-120.5	8
130539	E1965	South of Rio Guadalupe, Baja	32.1	-116.5	1
130540	E3144	Mission Bay Breakwater, San Diego, CA	32.8	-117.2	5
130541	E3144	Mission Bay Breakwater, San Diego, CA	32.8	-117.2	12
130542	E4390	Sixty miles South of San Felipe, Baja	30.2	-114.6	7
130543	E9930	Tres Marias, Baja	26	-112.1	1
130544	E9930	Tres Marias, Baja	26	-112.1	1
130545	E9930	Tres Marias, Baja	26	-112.1	1
130546	2403-	Santa Cruz, CA	37	-122	1
130547	A4230	Anaheim Landing, CA	33.7	-118.1	35
130548	2411-	Pigeon Point, CA	37.2	-122.4	47
130549	2388-	San Pedro, CA	33.7	-118.3	12
130550	3117-	CA	NA	NA	45
130551	D8919	Tiburon, CA	37.9	-122.5	18
130552	E97	Moss Beach, Half Moon Bay, CA	37.5	-122.5	10
130553	D8930	San Diego, CA	32.7	-117.2	1
130554	IP14572	Sunset Cliffs, Osprey Street, San Diego, CA	32.7	-117.3	3
130555	IP14539	Punta Abreojos, Baja	26.7	-113.6	18
130556	IP14594	Point Loma Lighthouse, CA	32.7	-117.2	1
130557	IP14594	Point Loma Lighthouse, CA	32.7	-117.2	2
130558	IP14594	Point Loma Lighthouse, CA	32.7	-117.2	3
130559	IP14597	Point Loma Lighthouse, CA	32.7	-117.2	1
130560	E3747	West side of Punta Banda, Baja	31.7	-116.7	8
130561	E6853	Cabrillo Beach, CA	33.7	-118.3	29
130562	E6852	Venice, CA	34	-118.5	52
130563	R7163	Port Crescent, WA	48.2	-123.7	2
130564	2910-	Port Harford, CA	35.2	-120.8	7
130565	3341-	Kirby Creek, Vancouver Island, BC	48.4	-123.9	5
130566	B358	Neah Bay, WA	48.4	-124.6	4
130567	E6890	Pacific Grove, CA	36.6	-121.9	4
130568	E6844	Timber Cove, CA	38.5	-123.3	10
130569	E67	Carmet, CA	38.4	-123.1	4
130570	2392-	San Diego, CA	32.7	-117.2	8
130571	E197	Near CA-OR border, Smith River, CA	41.9	-124.2	4
130572	E3143	Mission Bay Breakwater, San Diego, CA	32.8	-117.2	5
130573	E49	Dillon Beach, CA	38.3	-123	5
130574	3342-	Clo-oose, Vancouver Island, BC	48.7	-124.8	5
130575	3122-	OR	NA	NA	6
130576	E772	Mussel Point, CA	38.3	-123.1	3
130577	E628	East Point, Santa Rosa Island, CA	33.9	-120	3
130578	E387	One mile north of San Simeon, CA	35.6	-121.2	19
130579	2403-	Santa Cruz, CA	37	-122	39
130580	D8919	Moss Beach, Half Moon Bay, CA	37.5	-122.5	14
130581	E644	Gaviota, CA	34.5	-120.2	19
130582	E6845	Crescent Beach, WA	48.7	-122.9	4
130583	E214	North of Redwood Creek, Orick, CA	41.3	-124.1	7
130584	E402	Brown Island, Friday Harbor, WA	48.5	-123	6
130585	E637	Rincon Beach, CA	34.3	-119.4	10
130586	E637	Rincon Beach, CA	34.3	-119.4	1
130587	E637	Rincon Beach, CA	34.3	-119.4	1
130588	R3434	Victoria, BC	48.4	-123.4	3
130589	R3453	Corona del Mar, CA	33.6	-117.9	2
130590	2412-	Bolinas Bay, CA	37.9	-122.7	2
130591	E1998	Point Loma, CA	32.7	-117.2	20
130592	R7174	Restoration Point, Bainbridge Island, WA	47.6	-122.5	2
130593	E811	Seal Beach rocks near Lighthouse Point, CA	38.9	-123.7	4
130595	E507	Moss Landing, CA	36.8	-121.8	22
130596	E6848	Neah Bay, WA	48.4	-124.6	2
130597	E755	Santa Cruz, CA	37	-122	5
130598	E891	Pigeon Point, CA	37.2	-122.4	9
130599	E177	Russian Gulch, CA	39.3	-123.8	5
130600	E586	Southeast corner of San Nicolas Island, CA	33.2	-119.5	12
130601	E191	North end of False Klamath Cove, Requia, CA	41.6	-124.1	4
130602	E1309	Davis Bay, Lopez Island, WA	48.5	-122.9	3
130603	E744	Horseshoe Cove, CA	38.3	-123.1	4
130604	2419-	Moss Beach, Half Moon Bay, CA	37.5	-122.5	33
130605	2388-	San Pedro, CA	33.7	-118.3	8
130606	R1685	Golden Gate, CA	37.8	-122.5	127
130607	2781-	Crystal Pier, Pacific Beach, CA	32.8	-117.3	19
130608	2439-	Bolinas, CA	37.9	-122.7	3
130609	2439-	Bolinas, CA	37.9	-122.7	3
130610	2439-	Bolinas, CA	37.9	-122.7	19
130611	R1744	Russian Gulch, CA	39.3	-123.8	40
130612	E3166	Dana Point, CA	33.5	-117.7	5
130613	R3448	Capitan, CA	34.5	-120	10
130614	IP10950	San Nicolas Island, CA	33.2	-119.5	1
130615	E1458	Just north of Scripps, La Jolla, CA	32.9	-117.3	10
130616	E6849	Rosario Beach, WA	48.4	-122.7	1
130617	E358	Horseshoe Point, CA	38.6	-123.4	2
130618	E659	One mile north of Pigeon Point, CA	37.2	-122.4	1
130619	E1327	Brown Island, Friday Harbor, WA	48.5	-123	2
130620	2392-	San Diego, CA	32.7	-117.2	70
130621	2391-	Santa Barbara Island, CA	33.5	-119	10
130622	R1696	Lagoon Head, Baja	NA	NA	23
130623	R1671	Bahia Tortugas, Baja	27.7	-114.9	7
130624	E6102	Mussel Point, CA	38.3	-123.1	35
130625	E5059	Russian Gulch, CA	39.3	-123.8	2
130626	E869	Frazer Point, Santa Cruz Island, CA	34	-119.8	1
130627	E702	Cayucos, CA	35.4	-120.9	10
130628	E147	Spanish Bay, 17 Mile Drive, Monterey, CA	36.6	-121.9	23
130629	E2120	Bodega Head, CA	38.3	-123.1	11
130630	E580	South-southeast shore, Santa Barbara Island, CA	33.5	-119	7
130631	D8919	Mussel Point, CA	38.3	-123.1	2
130632	A4217	Stinson Beach, CA	37.9	-122.6	16
130634	E677	Whites Point, San Pedro Qd, CA	33.7	-118.3	9
130635	E2581	Bird Rock, CA	32.8	-117.3	10
130636	E1299	Minnesota Reef, San Juan Island, WA	48.5	-123	2
130637	E1255	Soberanes Point, CA	36.4	-121.9	3
130638	E195	South end of False Klamath Cove, Requia, CA	41.6	-124.1	2
130639	2770-	San Juan Island, WA	48.5	-123.1	17
130640	2390-	Santa Barbara, CA	34.4	-119.7	52
130641	E3904	Five miles north of Rosarita Beach, Baja	32.4	-117.1	35
130643	E153	Tomales Point, CA	38.2	-123	11
130645	E307	Mission Point, CA	36.6	-121.9	4
130646	E733	Shell Beach, Bodega Head, CA	38.4	-123.1	1
130647	E2518	Solana Beach, Del Mar, CA	33	-117.3	3
130648	E342	Drakes Estero, CA	38	-122.9	2
130649	E665	Scott Creek, Davenport, CA	37	-122.2	14
130650	E170	Duncans Point, CA	38.4	-123.1	4
130651	E819	North of Lighthouse Point, Arena, CA	39	-123.7	2
130652	E717	Salt Point, CA	38.6	-123.3	1
130653	2390-	Santa Barbara, CA	34.4	-119.7	1
130654	E6521	Morro Beach, CA	35.4	-120.9	13
130656	E222	Two miles south of Cape Mendocino, CA	40.4	-124.4	4
130658	2419-	Moss Beach, Half Moon Bay, CA	37.5	-122.5	4
130659	E357	Horseshoe Point, CA	38.6	-123.4	6
130660	2395-	Monterey, CA	36.6	-121.9	31
130661	2439-	Bolinas, CA	37.9	-122.7	29
130662	E6838	Monterey, CA	36.6	-121.9	3
130663	3117-	CA	NA	NA	5
130664	E258	Near mouth of Elk Creek, South of Elk Creek, CA	39.1	-123.7	1
130665	E743	Horseshoe Cove, CA	38.3	-123.1	1
130666	E1239	Pigeon Point, CA	37.2	-122.4	3
130667	E3828	West of Canada Tecelate, Santa Rosa Island, CA	34	-120.1	16
130668	E266	Havens Neck, CA	38.8	-123.6	1
130669	E1996	Point Loma, CA	32.7	-117.2	1
130670	E1959	South of Rio Guadalupe, Baja	32.1	-116.5	3
130671	E386	One mile south of San Simeon, CA	35.6	-121.2	5
130672	E1578	Arena Cove, CA	38.9	-123.7	1
130673	E243	Cape Vizcaino, CA	39.7	-123.8	6
130675	E442	Dana Point, CA	33.5	-117.7	27
130676	E818	Point Arena, CA	38.9	-123.7	2
130677	B831	Ocean Beach, CA	37.8	-122.5	7
130678	B911	Cambria, CA	35.6	-121.1	4
130679	2412-	Bolinas Bay, CA	37.9	-122.7	2
130680	E6839	San Simeon, CA	35.6	-121.2	1
130681	E754	Santa Cruz, CA	37	-122	2
130682	E517	San Simeon, CA	35.6	-121.2	1
130683	E1399	One and a half miles north of Dana Point, CA	33.5	-117.7	4
130684	E490	Rincon Beach, CA	34.3	-119.4	3
130685	E3248	South of La Mision River, Baja	32.1	-116.9	3
130686	E490	Rincon Beach, CA	34.3	-119.4	2
130687	E48	Dillon Beach, CA	38.3	-123	1
130688	E983	Devils Slide, San Diego, CA	32.9	-117.3	1
130689	E374	Government Point, CA	34.4	-120.5	5
130690	E37	Duxbury Reef, CA	37.9	-122.7	4
130691	E96	Moss Beach, Half Moon Bay, CA	37.5	-122.5	12
130692	E3835	Half mile north of Point Buchon, CA	35.3	-120.9	2
130693	E146	Monterey, CA	36.6	-121.9	4
130694	E1973	Halfway Reef, Baja	0	0	7
130695	E4024	San Quintin Bay, Baja	30.6	-115.9	1
130696	E627	East Point, Santa Rosa Island, CA	33.9	-120	1
130697	2403-	Santa Cruz, CA	37	-122	7
130698	2390-	Santa Barbara, CA	34.4	-119.7	3
130699	2400-	Puget Sound, WA	47.8	-122.4	4
130700	2412-	Bolinas Bay, CA	37.9	-122.7	2
130701	2411-	Pigeon Point, CA	37.2	-122.4	28
130702	2410-	Half Moon Bay, CA	37.5	-122.4	18
130703	R1652	Stinson Beach, CA	37.9	-122.6	9
130704	2398-	Straits of Fuca, Vancouver Island, CA	48.3	-124	1
130705	R1775	Point Pinos, Pacific Grove, CA	36.6	-121.9	5
130706	2439-	Bolinas, CA	37.9	-122.7	3
130707	2432-	Port Townsend, WA	48.1	-122.8	1
130708	E1949	South Coronado Island, Baja	32.4	-117.2	2
130709	E6508	1000 yards south of Cape Mendocino, CA	40.4	-124.4	3
130710	R3398	Friday Harbor, WA	48.5	-123	3
130711	2391-	Santa Barbara Island, CA	33.5	-119	6
130712	2896-	Sitka, AK	57.1	-135.3	3
130713	E720	Salt Point, CA	38.6	-123.3	3
130714	2395-	Monterey, CA	36.6	-121.9	1
130715	R516	Bear Bay, Baranoff Island, AK	57.5	-135.5	1
130716	R517	Red Bluff Bay, Baranoff Island, AK	56.9	-134.8	1
130717	2440-	Tobin Beach, CA	0	0	3
130718	B892	Friday Harbor, WA	48.5	-123	4
130719	R7163	Port Crescent, WA	48.2	-123.7	1
130720	R7209	Pillar Point near Pysht, WA	48.2	-124.1	3
130721	R7173	Clallam Bay, WA	48.3	-124.3	3
130722	3341-	Kirby Creek, Vancouver Island, BC	48.4	-123.9	3
130723	B835	Coronado, CA	32.7	-117.2	5
130724	B7358	Chatham, AK	57.5	-134.9	1
130725	A9119	Punta Santo Tomas, Baja	31.6	-116.7	2
130726	A6601	Moss Beach, Half Moon Bay, CA	37.5	-122.5	1
130727	R3385	Kodiak, AK	57.8	-152.4	2
130728	A3991	Newport Beach, CA	33.6	-117.9	14
130729	E745	Horseshoe Cove, CA	38.3	-123.1	1
130730	R7210	Freshwater Bay, WA	48.1	-123.6	13
130731	E1863	Tijuana River, CA-Baja border	32.6	-117.1	2
130732	E893	Pigeon Point, CA	37.2	-122.4	11
130733	E736	Shell Beach, Bodega Head, CA	38.4	-123.1	2
130734	2395-	Monterey, CA	36.6	-121.9	2
130735	2395-	Monterey, CA	36.6	-121.9	3
130736	R1637	East coast of Graham Island, BC	53.4	-131.9	5
130737	R1583	False Narrows, Unimak Island, AK	54.8	-163.4	2
130739	R7162	Twin Rivers, WA	48.2	-124	3
130740	E1262	Del Mar Ranch, CA	38.7	-123.5	4
130741	R3385	Kodiak, AK	57.8	-152.4	1
130742	E5060	Russian Gulch, CA	39.3	-123.8	2
130744	E3837	Half mile north of Point Buchon, CA	35.3	-120.9	1
130745	E403	Brown Island, Friday Harbor, WA	48.5	-123	3
130746	E2122	Bodega Head, CA	38.3	-123.1	2
130747	E425	Del Mar Point, CA	38.7	-123.5	4
130748	2392-	San Diego, CA	32.7	-117.2	5
130749	E2455	Coal Oil Point, Galeta, CA	34.4	-119.9	2
130750	E1687	South Coronado Island, Baja	32.4	-117.2	7
130751	E1908	South Coronado Island, Baja	32.4	-117.2	9
130752	E6884	Two miles west of Point Pinos, CA	36.6	-121.9	2
130753	E614	San Miguel Island, Santa Barbara, CA	34	-120.4	1
130754	E6885	Moss Beach, Half Moon Bay, CA	37.5	-122.5	8
130755	D8919	Half Moon Bay, CA	37.5	-122.4	2
130756	E247	One mile south of Hardy, CA	39.7	-123.8	3
130757	E1936	South Coronado Island, Baja	32.4	-117.2	13
130758	E1799	Point Loma, CA	32.7	-117.2	5
130759	E289	Point Pinos, Pacific Grove, CA	36.6	-121.9	1
130760	E1779	Point Loma, CA	32.7	-117.2	12
130761	E1640	Santo Tomas, Baja	31.6	-116.4	2
130762	E6880	San Simeon, CA	35.6	-121.2	1
130763	E890	Arena Cove, CA	38.9	-123.7	6
130764	E1846	Point Loma, CA	32.7	-117.2	3
130765	E6887	Shell Beach, Bodega Head, CA	38.4	-123.1	2
130766	E227	Two miles south of Cape Mendocino, CA	40.4	-124.4	9
130767	E1823	Point Loma, CA	32.7	-117.2	6
130768	2395-	Monterey, CA	36.6	-121.9	2
130769	E1856	Off Tijuana River, Imperial Beach, CA	32.6	-117.1	1
130770	E1533	Garrison Bay, San Juan Island, WA	48.6	-123.2	7
130771	E5050	Russian Gulch, CA	39.3	-123.8	5
130772	E1878	Off Tijuana River, Imperial Beach, CA	32.6	-117.1	17
130773	E1892	Off Tijuana River, Imperial Beach, CA	32.6	-117.1	10
130774	E1674	North Coronado Island, Baja	32.4	-117.3	10
130775	E6882	Arch Rock, CA	38	-122.8	7
130776	E1681	North Coronado Island, Baja	32.4	-117.3	1
130777	E649	Pillar Point, Half Moon Bay, CA	37.5	-122.5	1
130778	E6886	Ensenada, Baja	31.9	-116.6	2
130779	E135	Moss Beach, Half Moon Bay, CA	37.5	-122.5	9
130780	2787-	Fort Bragg, CA	39.4	-123.8	20
130781	A9119	Punta Santo Tomas, Baja	31.6	-116.7	2
130782	E2463	Point Loma, CA	32.7	-117.2	5
130783	2395-	Monterey, CA	36.6	-121.9	5
130784	2393-	San Francisco, CA	37.8	-122.4	7
130786	2392-	San Diego, CA	32.7	-117.2	10
130794	2774-	Round Island, Baja	32.4	-117.2	6
130812	2485-	Pescadero, CA	37.3	-122.4	19
130813	E101	Moss Beach, Half Moon Bay, CA	37.5	-122.5	3
130814	R1668	San Diego, CA	32.7	-117.2	35
130815	E6933	Shell Beach, Bodega Head, CA	38.4	-123.1	5
130816	E230	Two miles south of Cape Mendocino, CA	40.4	-124.4	8
130817	E829	Yankee Point, Soberones, CA	36.5	-121.9	10
130819	D8919	Moss Beach, Half Moon Bay, CA	37.5	-122.5	4
130820	E72	Carmet, CA	38.4	-123.1	18
130822	3394-	Victoria, BC	48.4	-123.4	7
130823	E6926	Arch Rock, CA	38	-122.8	7
130824	E1387	Two miles south of Scripps, La Jolla, CA	32.8	-117.3	6
130825	E597	Isthmus Cove, Santa Catalina Island, CA	33.4	-118.5	10
130826	2419-	Moss Beach, Half Moon Bay, CA	37.5	-122.5	36
130827	E1580	Arena Cove, CA	38.9	-123.7	7
130828	E895	Pigeon Point, CA	37.2	-122.4	16
130829	B841	Bird Rock, CA	32.8	-117.3	32
130830	E589	Southeast corner of San Nicolas Island, CA	33.2	-119.5	10
130831	B876	Mission Beach, CA	32.8	-117.3	38
130832	E3751	North side of Punta Banda, Baja	31.7	-116.7	16
130833	E140	Point Pinos, Pacific Grove, CA	36.6	-121.9	4
130834	E40	Duxbury Reef, CA	37.9	-122.7	10
130835	2891-	Stewart's Point, CA	38.7	-123.4	1
130836	E813	Seal Beach rocks near Lighthouse Point, CA	38.9	-123.7	16
130837	2412-	Bolinas Bay, CA	37.9	-122.7	6
130838	E430	Del Mar Point, CA	38.7	-123.5	2
130839	E309	Mission Point, CA	36.6	-121.9	4
130840	E760	Santa Cruz, CA	37	-122	4
130841	2410-	Half Moon Bay, CA	37.5	-122.4	6
130842	E578	Johnson's Landing, Santa Catalina Island, CA	33.5	-118.5	14
130843	E697	Morro Rock, CA	35.4	-120.9	1
130844	E346	Drakes Estero, CA	38	-122.9	6
130845	E3145	Mission Bay Breakwater, San Diego, CA	32.8	-117.2	3
130846	E724	Salt Point, CA	38.6	-123.3	1
130848	E871	Frazer Point, Santa Cruz Island, CA	34	-119.8	6
130849	E630	East Point, Santa Rosa Island, CA	33.9	-120	12
130850	E1264	Del Mar Ranch, CA	38.7	-123.5	4
130851	E3253	South of La Mision River, Baja	32.1	-116.9	4
130852	E3062	Punta Banda, Baja	31.7	-116.7	3
130853	E6925	Venice, CA	34	-118.5	10
130854	E3839	Half mile north of Point Buchon, CA	35.3	-120.9	9
130855	E391	One mile north of San Simeon, CA	35.6	-121.2	11
130856	E766	Russian Gulch, CA	39.3	-123.8	1
130857	E3170	Dana Point, CA	33.5	-117.7	16
130858	E2002	Point Loma, CA	32.7	-117.2	2
130859	E690	Gaviota Beach, CA	34.5	-120.2	2
130860	E6928	Morro Bay, CA	35.4	-120.8	2
130861	E2906	Halfway between Punta Mezquite and Punta Sal Si Puedes, Baja	32.1	-116.9	1
130862	E822	North of Lighthouse Point, Arena, CA	39	-123.7	5
130863	E2582	Bird Rock, CA	32.8	-117.3	3
130864	E251	One mile south of Hardy, CA	39.7	-123.8	7
130865	E1404	Monarch Bay, CA	33.5	-117.7	10
130866	E624	Willows Cove, Santa Cruz Island, CA	34	-119.8	11
130867	IP14539	Punta Abreojos, Baja	26.7	-113.6	1
130868	IP14591	Sunset Cliffs, San Diego, CA	32.7	-117.3	22
130869	IP14593	Flood Control Channel, San Diego, CA	32.8	-117.3	2
130870	IP14596	Sunset Cliffs, Hill Street, San Diego, CA	32.7	-117.3	6
130872	R1774	Carmel Bay, CA	36.5	-122	22
130873	B361	Clallam Bay, WA	48.3	-124.3	1
130874	E6883	Crescent Beach, WA	48.7	-122.9	2
130875	2403-	Santa Cruz, CA	37	-122	7
130876	2389-	Santa Catalina Island, CA	33.4	-118.4	5
131502	E3904	Five miles north of Rosarita Beach, Baja	32.4	-117.1	5
153640	B835	Coronado, CA	32.7	-117.2	8
157380	IP14766	Mission Bay Breakwater, San Diego, CA	32.8	-117.2	1
157394	IP14767	Five miles north of San Simeon, CA	35.7	-121.3	1
157395	IP14767	Five miles north of San Simeon, CA	35.7	-121.3	3
157406	IP14768	Hearst Park, CA	35.6	-121.2	3
157408	IP14768	Hearst Park, CA	35.6	-121.2	1
157410	IP14769	Co Parle North of Point Conception, CA	34.4	-120.5	1
157411	IP14769	North Point Conception, Santa Barbara, CA	34.4	-120.5	2
157419	IP14771	National Forest Beach, Cape San Martin, CA	35.9	-121.5	5
157421	IP14771	National Forest Beach, Cape San Martin, CA	35.9	-121.5	1
157423	IP14771	National Forest Beach, Cape San Martin, CA	35.9	-121.5	4
157424	IP14771	National Forest Beach, Cape San Martin, CA	35.9	-121.5	2
157427	IP14772	Foot of Jolon Road, CA	36	-121.5	11
157429	IP14773	Sunset Cliffs, Osprey Street, San Diego, CA	32.7	-117.3	3
157460	IP14781	Sunset Cliffs, Granger Street, San Diego, CA	32.7	-117.3	3
157467	IP14787	Shell Beach, La Jolla, CA	32.8	-117.3	9
157470	IP14802	Mission Bay Breakwater, San Diego, CA	32.8	-117.2	10
157490	IP14794	Five miles north of Doheny Beach, CA	33.5	-117.7	3
157685	IP14861	Half mile south of Mussel Point, CA	38.3	-123.1	1
157686	IP14861	Half mile south of Mussel Point, CA	38.3	-123.1	2
157688	IP14862	Bodega Bay, CA	38.3	-123	1
157697	IP14869	Mussel Point, CA	38.3	-123.1	2
157698	IP14869	Mussel Point, CA	38.3	-123.1	1
A list of UCMP lots, with associated UCMP locality numbers, locality names, latitudinal and longitudinal coordinates, and the number of individuals in each lot. Coordinates have been rounded to the nearest 0.1 degree. Note that ‘number of individuals’ refers to the number of objects that have been successfully processed 2D morphometric measurements, and may not reflect the total number of individuals present in each full-lot overview image.					

**Table 2:** Number of species per site, with locality strings and species names

**UCMP specimen number**	**locality string**	**latitude**	**longitude**	**number of species**	**species**
53648, 53679, 53681, 130188, 130196, 130243, 130244, 130694	Halfway Reef, Baja			5	*L. fenestrata, L. digitalis, L. pelta, L. paradigitalis, L. asmi*
53142, 53387, 130326, 130338, 130717	Tobin Beach, CA			4	*L. digitalis, L. pelta, L. scutum, A. mitra*
130125	Cabo San Lucas, Baja	22.9	-109.9	1	*L. atrata*
53594, 53611	La Paz, Baja	24.1	-110.3	1	*L. mesoleuca*
130543, 130544, 130545	Tres Marias, Baja	26	-112.1	3	*L. dalliana, L. digitalis, L. paradigitalis*
53538, 130555, 130867	Punta Abreojos, Baja	26.7	-113.6	3	*L. fenestrata, L. digitalis, L. scabra*
53680, 130186, 130187	South of Punta Abreojos, Baja	26.8	-113.5	2	*L. digitalis, L. paradigitalis*
53095, 53204, 130170, 130313, 130421, 130422, 130623	Bahia Tortugas, Baja	27.7	-114.9	6	*L. limatula, T. testudinalis, L. digitalis, L. gigantea, L. scutum, L. persona*
130123	Estero de Bahia de Todas Santos, Baja	27.7	-114.9	1	*L. paleacea*
53083	North Cedros Island, Baja	28.1	-115.2	1	*L. limatula*
53586	Los Angeles Bay, Baja	28.9	-113.6	1	*L. mesoleuca*
130310	Barracks Beach, Baja	29	-118.3	1	*L. gigantea*
53050, 130305	Isla Guadalupe, Baja	29	-118.3	2	*L. limatula, L. gigantea*
130304	The Nursery, Isla Guadalupe, Baja	29	-118.3	1	*L. gigantea*
53571, 53582, 53593, 130281	San Luis Gonzaga Bay, Baja	29.8	-114.4	2	*L. dalliana, L. mesoleuca*
130106	Punta Final, Baja	29.8	-114.3	1	*L. dalliana*
53669	Punta Baja, Baja	29.9	-115.8	1	*L. paradigitalis*
53572	Twenty-eight miles south of Puertecitos, Baja	30	-114.5	1	*L. dalliana*
53577, 53588	Nineteen miles south of Puertecitos, Baja	30.1	-114.6	2	*L. stanfordiana, L. dalliana*
130542	Sixty miles south of San Felipe, Baja	30.2	-114.6	1	*L. dalliana*
53562, 53574, 53589	Five miles south of Puertecitos, Baja	30.3	-114.7	2	*L. mesoleuca, L. dalliana*
53564	One mile south of Puertecitos, Baja	30.3	-114.6	1	*L. dalliana*
53557, 53575, 53583, 53605, 53606	Puertecitos, Baja	30.3	-114.6	2	*L. dalliana, L. stanfordiana*
53569, 53591, 130122	Gulf of CA	30.3	-113.7	2	*L. mesoleuca, L. discus*
53560	Just north of Puertecitos, Baja	30.4	-114.6	1	*L. dalliana*
53584	Two miles north of Puertecitos, Baja	30.4	-114.6	1	*L. stanfordiana*
53639, 130695	San Quintin Bay, Baja	30.6	-115.9	2	*L. paradigitalis, L. asmi*
53578, 53598, 53615, 130126	San Felipe, Baja	31	-114.8	3	*L. concreta, L. mesoleuca, A. turveri*
130309, 130725, 130781	Punta Santo Tomas, Baja	31.6	-116.7	2	*L. gigantea, A. mitra*
53159, 53418, 53492, 130761	Santo Tomas, Baja	31.6	-116.4	4	*L. digitalis, L. pelta, L. insessa, A. mitra*
53315, 53326	Blowhole west of Bauda Fish Camp, Baja	31.7	-116.7	1	*L. pelta*
53030, 53542, 53549, 130108, 130195	Estero de Punta Banda, Baja	31.7	-116.7	3	*L. limatula, L. depicta, L. paradigitalis*
53155, 130832	North side of Punta Banda, Baja	31.7	-116.7	2	*L. digitalis, L. scabra*
53044, 53051, 53062, 53091, 53179, 53637, 130317, 130852	Punta Banda, Baja	31.7	-116.7	5	*L. limatula, L. digitalis, L. paradigitalis, L. gigantea, L. scabra*
53374, 130312, 130316	South side of Punta Banda, Baja	31.7	-116.7	2	*L. pelta, L. gigantea*
130560	West side of Punta Banda, Baja	31.7	-116.7	1	*L. digitalis*
53088, 130102, 130105, 130395, 130778	Ensenada, Baja	31.9	-116.6	3	*L. limatula, L. persona, A. mitra*
53036	Rio Guadalupe, Baja	32	-116.5	1	*L. limatula*
53488, 130321, 130861	Halfway between Punta Mezquite and Punta Sal Si Puedes, Baja	32.1	-116.9	3	*L. insessa, L. gigantea, L. scabra*
53017	La Mision River, Baja	32.1	-116.9	1	*L. limatula*
53005, 53022, 53163, 130194, 130322	Punta Piedra, Baja	32.1	-116.9	4	*L. limatula, L. digitalis, L. paradigitalis, L. gigantea*
53182, 53651, 130311, 130685, 130851	South of La Mision River, Baja	32.1	-116.9	5	*L. digitalis, L. fenestrata, L. gigantea, L. asmi, L. scabra*
53644, 130233, 130296, 130539, 130670	South of Rio Guadalupe, Baja	32.1	-116.5	4	*L. fenestrata, L. gigantea, L. scabra, L. asmi*
53641	Rosarita Beach, Baja	32.3	-117.1	1	*L. paradigitalis*
130282	Middle Coronados Island, Baja	32.4	-117.3	1	*T. rosacea*
130774, 130776	North Coronado Island, Baja	32.4	-117.3	1	*A. mitra*
53415, 130794	Round Island, Baja	32.4	-117.2	2	*L. pelta, L. scabra*
130190, 130191, 130708, 130750, 130751, 130757	South Coronado Island, Baja	32.4	-117.2	3	*L. scabra, A. funiculata, A. mitra*
130110, 130111	Southeast Coronado Island, Los Coronados, Baja	32.4	-117.2	1	*A. funiculata*
53676, 130248, 130318, 130507, 130641, 131502	Five miles north of Rosarita Beach, Baja	32.4	-117.1	6	*L. limatula, L. fenestrata, L. gigantea, L. scabra, L. digitalis, L. asmi*
53216	Mouth of Tijuana River, Imperial Beach, CA	32.6	-117.1	1	*T. rosacea*
130284	Off mouth of Tijuana River, Imperial Beach, CA	32.6	-117.1	1	*T. rosacea*
130769, 130772, 130773	Off Tijuana River, Imperial Beach, CA	32.6	-117.1	1	*A. mitra*
130731	Tijuana River, CA-Baja border	32.6	-117.1	1	*A. mitra*
53381, 130677	Ocean Beach, CA	32.7	-117.3	2	*L. pelta, L. asmi*
53465, 157460	Sunset Cliffs, Granger Street, San Diego, CA	32.7	-117.3	2	*L. insessa, L. pelta*
53643, 130466, 130870	Sunset Cliffs, Hill Street, San Diego, CA	32.7	-117.3	3	*L. fenestrata, L. gigantea, L. scabra*
130554, 157429	Sunset Cliffs, Osprey Street, San Diego, CA	32.7	-117.3	2	*L. digitalis, L. scabra*
53059, 130460, 130868	Sunset Cliffs, San Diego, CA	32.7	-117.3	3	*L. limatula, L. gigantea, L. scabra*
53494	100 m south of San Diego, 20 m offshore, CA	32.7	-117.2	1	*L. insessa*
130518, 130519, 130520	Coronado Island, CA	32.7	-117.2	3	*L. scabra, L. digitalis, A. mitra*
53019, 53119, 53227, 53411, 130465, 130723, 153640	Coronado, CA	32.7	-117.2	6	*L. limatula, L. digitalis, L. pelta, L. gigantea, A. mitra, L. scutum*
53217, 53311, 53312, 53321, 130379, 130467, 130556, 130557, 130558, 130559	Point Loma Lighthouse, CA	32.7	-117.2	5	*L. pelta, L. persona, L. gigantea, L. digitalis, L. paradigitalis*
53061, 53206, 53207, 53215, 53489, 53491, 53667, 130591, 130669, 130758, 130760, 130764, 130767, 130782, 130858	Point Loma, CA	32.7	-117.2	8	*L. limatula, T. rosacea, L. insessa, L. fenestrata, L. digitalis, L. asmi, A. mitra, L. scabra*
53042, 53057, 53229, 53536, 53654, 130148	San Diego Jetty, San Diego, CA	32.7	-117.2	4	*L. limatula, L. pelta, L. depicta, L. digitalis*
53037, 53043, 53046, 53056, 53058, 53060, 53063, 53064, 53065, 53066, 53131, 53153, 53166, 53222, 53280, 53495, 53535, 53541, 53547, 53646, 53661, 53662, 130128, 130129, 130146, 130155, 130158, 130211, 130269, 130285, 130286, 130287, 130306, 130451, 130452, 130481, 130526, 130553, 130570, 130620, 130748, 130786, 130814	San Diego, CA	32.7	-117.2	12	*L. limatula, L. digitalis, L. pelta, L. insessa, L. depicta, L. fenestrata, L. persona, L. scutum, T. rosacea, L. gigantea, L. scabra, A. mitra*
53087, 53625, 130458, 130464, 130635, 130829, 130863	Bird Rock, CA	32.8	-117.3	4	*L. limatula, L. gigantea, L. digitalis, L. scabra*
130607	Crystal Pier, Pacific Beach, CA	32.8	-117.3	1	*L. digitalis*
53546, 53548, 53650, 53668, 130514, 130869	Flood Control Channel, San Diego, CA	32.8	-117.3	4	*L. depicta, L. fenestrata, L. limatula, L. scabra*
53021, 53137, 53545, 53675, 130442, 130468	La Jolla, CA	32.8	-117.3	6	*L. limatula, L. digitalis, L. depicta, L. scutum, L. persona, L. gigantea*
53473, 130831	Mission Beach, CA	32.8	-117.3	2	*L. insessa, L. scabra*
53493	Pacific Beach, CA	32.8	-117.3	1	*L. insessa*
53375, 157467	Shell Beach, La Jolla, CA	32.8	-117.3	2	*L. pelta, L. insessa*
130300, 130824	Two miles south of Scripps, La Jolla, CA	32.8	-117.3	2	*L. gigantea, L. scabra*
53038, 130189, 130540, 130541, 130572, 130845, 157380, 157470	Mission Bay Breakwater, San Diego, CA	32.8	-117.2	5	*L. limatula, L. paradigitalis, L. digitalis, L. scabra, L. fenestrata*
53029, 53034, 53544, 53638	Mission Bay, CA	32.8	-117.2	3	*L. limatula, L. depicta, L. scutum*
53077	Northwest Mission Bay, San Diego, CA	32.8	-117.2	1	*L. limatula*
53054, 53105, 53165	San Clemente Island, CA	32.9	-118.5	2	*L. limatula, L. digitalis*
130688	Devils Slide, San Diego, CA	32.9	-117.3	1	*L. asmi*
130615	Just north of Scripps, La Jolla, CA	32.9	-117.3	1	*L. digitalis*
130302	North of Scripps, La Jolla, CA	32.9	-117.3	1	*L. gigantea*
130103	South of Scripps, La Jolla, CA	32.9	-117.3	1	*L. limatula*
53011, 53490, 130193, 130647	Solana Beach, Del Mar, CA	33	-117.3	4	*L. limatula, L. insessa, L. fenestrata, L. digitalis*
53148, 53258, 53421, 53468, 130614	San Nicolas Island, CA	33.2	-119.5	3	*L. digitalis, L. pelta, L. insessa*
53302, 53539, 130496, 130600, 130830	Southeast corner of San Nicolas Island, CA	33.2	-119.5	5	*A. strigatella, L. fenestrata, L. gigantea, L. digitalis, L. scabra*
130240, 130825	Isthmus Cove, Santa Catalina Island, CA	33.4	-118.5	2	*L. fenestrata, L. scabra*
130471	Moonstone Beach, Santa Catalina Island, CA	33.4	-118.4	1	*L. gigantea*
53089, 53128, 130876	Santa Catalina Island, CA	33.4	-118.4	2	*L. limatula, L. digitalis*
53232	Off of the south-southeast shore of Santa Barbara Island, CA	33.5	-119	1	*L. pelta*
53007, 53093, 53439, 130172, 130173, 130174, 130450, 130491, 130621, 130711	Santa Barbara Island, CA	33.5	-119	7	*L. limatula, L. pelta, L. digitalis, L. scabra, L. persona, L. gigantea, A. mitra*
130630	South-southeast shore, Santa Barbara Island, CA	33.5	-119	1	*L. digitalis*
53647, 130842	Johnson's Landing, Santa Catalina Island, CA	33.5	-118.5	2	*L. fenestrata, L. scabra*
53395	Laguna Beach, CA	33.5	-117.8	1	*L. pelta*
53476	Laguna Tide Pools, Laguna Beach, CA	33.5	-117.8	1	*L. insessa*
53023, 53092, 53147, 53234, 53398, 53443, 53632, 53633, 53634, 53645, 130308, 130527, 130612, 130675, 130857	Dana Point, CA	33.5	-117.7	8	*L. limatula, L. digitalis, L. pelta, L. insessa, L. scabra, L. fenestrata, L. gigantea, L. asmi*
157490	Five miles north of Doheny Beach, CA	33.5	-117.7	1	*L. pelta*
53169, 130259, 130307	Half mile north of Dana Point, CA	33.5	-117.7	3	*L. digitalis, L. fenestrata, L. gigantea*
53078, 53485, 130865	Monarch Bay, CA	33.5	-117.7	3	*L. limatula, L. insessa, L. scabra*
130683	One and a half miles north of Dana Point, CA	33.5	-117.7	1	*L. asmi*
130589	Corona del Mar, CA	33.6	-117.9	1	*L. digitalis*
130301	Little Corona Beach, Corona del Mar, CA	33.6	-117.9	1	*L. gigantea*
130038	Newport Bay, CA	33.6	-117.9	1	*L. limatula*
53053, 53117, 53378, 53497, 130461, 130728	Newport Beach, CA	33.6	-117.9	6	*L. limatula, L. digitalis, L. pelta, L. insessa, L. gigantea, A. mitra*
53101	Newport Harbor, Newport Beach, CA	33.6	-117.9	1	*L. limatula*
53110, 53303, 53487, 130561	Cabrillo Beach, CA	33.7	-118.3	4	*L. limatula, L. pelta, L. insessa, L. digitalis*
53478	Los Angeles, CA	33.7	-118.3	1	*L. insessa*
53000, 53123, 53238	Point Fermin, CA	33.7	-118.3	3	*L. limatula, L. digitalis, L. pelta*
53018, 53045, 53115, 53219, 53444, 53474, 53543, 130101, 130327, 130446, 130530, 130549, 130605	San Pedro, CA	33.7	-118.3	8	*L. limatula, L. digitalis, L. pelta, L. insessa, L. depicta, L. scutum, L. persona, L. scabra*
53025, 53498, 130525, 130634	Whites Point, San Pedro Qd, CA	33.7	-118.3	4	*L. limatula, L. insessa, L. scabra, L. digitalis*
53109	Anaheim Bay, CA	33.7	-118.1	1	*L. limatula*
53094, 130547	Anaheim Landing, CA	33.7	-118.1	2	*L. limatula, L. digitalis*
130463	Lunada Bay, CA	33.8	-118.4	1	*L. gigantea*
53049, 53472, 53499, 53627, 53628	Palos Verdes Point, Redondo Beach, CA	33.8	-118.4	3	*L. limatula, L. insessa, L. scabra*
53396, 130577, 130696, 130849	East Point, Santa Rosa Island, CA	33.9	-120	4	*L. pelta, L. digitalis, L. asmi, L. scabra*
53102, 53145, 53245, 53423, 130109, 130293, 130753	San Miguel Island, Santa Barbara, CA	34	-120.4	6	*L. limatula, L. digitalis, L. pelta, L. scabra, L. gigantea, A. mitra*
53146, 130251	South end of Cuyler Harbor, San Miguel Island, CA	34	-120.4	2	*L. digitalis, L. fenestrata*
53262	Garedon Canyon, Santa Rosa Island, CA	34	-120.1	1	*L. pelta*
53111, 53250, 53481, 130502	Santa Rosa Island, Santa Barbara, CA	34	-120.1	4	*L. digitalis, L. pelta, L. insessa, L. gigantea*
130294, 130667	West of Canada Tecelate, Santa Rosa Island, CA	34	-120.1	2	*L. gigantea, L. asmi*
130454, 130626, 130848	Frazer Point, Santa Cruz Island, CA	34	-119.8	3	*L. gigantea, L. digitalis, L. scabra*
53144, 53260	Pelican Bay, Santa Cruz Island, CA	34	-119.8	2	*L. digitalis, L. pelta*
53152, 53389, 130134, 130411	Santa Cruz Island, CA	34	-119.8	3	*L. digitalis, L. pelta, L. persona*
53678	Scorpion Cove, Santa Cruz Island, CA	34	-119.8	1	*L. digitalis*
53075, 53132, 130114	Willows Anchorage, Santa Cruz Island, CA	34	-119.8	3	*L. limatula, L. digitalis, L. scabra*
53551, 130866	Willows Cove, Santa Cruz Island, CA	34	-119.8	2	*L. paradigitalis, L. scabra*
53660, 130517	North Anacapa Island, CA	34	-119.4	2	*L. fenestrata, L. scabra*
53192, 53554	South Anacapa Island, Ventura, CA	34	-119.4	2	*L. digitalis, L. fenestrata*
53379, 130242	East side of Point Dume, CA	34	-118.8	2	*L. pelta, L. fenestrata*
53097, 53228, 53479, 53480, 130192, 130562, 130853	Venice, CA	34	-118.5	6	*L. limatula, L. pelta, L. insessa, L. paradigitalis, L. digitalis, L. scabra*
53649, 130252, 130500, 130531, 130585, 130586, 130587, 130684, 130686	Rincon Beach, CA	34.3	-119.4	6	*L. fenestrata, L. gigantea, L. scabra, L. paradigitalis, L. digitalis, L. asmi*
157410	Co Parle North of Point Conception, CA	34.4	-120.5	1	*L. scabra*
53006, 53113, 130260, 130482, 130538, 130689	Government Point, CA	34.4	-120.5	6	*L. limatula, L. digitalis, L. fenestrata, L. gigantea, L. scabra, L. asmi*
53525, 157411	North Point Conception, Santa Barbara, CA	34.4	-120.5	2	*L. instabilis, L. limatula*
130749	Coal Oil Point, Galeta, CA	34.4	-119.9	1	*A. mitra*
53020, 53386, 53550, 130119, 130124, 130447, 130449, 130479, 130495, 130640, 130653, 130698	Santa Barbara, CA	34.4	-119.7	8	*L. limatula, L. pelta, L. depicta, T. rosacea, L. persona, L. gigantea, L. digitalis, L. asmi*
130258	Carpinteria, CA	34.4	-119.5	1	*L. fenestrata*
53254, 53331, 53482, 53483, 53565, 130256, 130299, 130859	Gaviota Beach, CA	34.5	-120.2	6	*L. pelta, L. insessa, L. dalliana, L. fenestrata, L. gigantea, L. scabra*
130581	Gaviota, CA	34.5	-120.2	1	*L. digitalis*
53114, 130254, 130255	Three miles east of Gaviota, CA	34.5	-120.2	3	*L. digitalis, L. fenestrata, L. paradigitalis*
130613	Capitan, CA	34.5	-120	1	*L. digitalis*
53190	Diablo Rock, CA	35.2	-120.9	1	*L. pelta*
53067, 53178, 53231, 53369, 130253	Intake Cove, Diablo Canyon Power Plant, CA	35.2	-120.9	5	*L. limatula, L. digitalis, L. pelta, L. insessa, L. fenestrata*
53671	North Diablo Cove, San Luis Obispo, CA	35.2	-120.9	1	*L. digitalis*
53672, 53682	South Diablo Cove, San Luis Obispo, CA	35.2	-120.9	2	*L. digitalis, L. pelta*
53670	Nobi Point, Port San Luis, CA	35.2	-120.8	1	*L. digitalis*
53052, 53364, 130564	Port Harford, CA	35.2	-120.8	3	*L. limatula, L. pelta, L. digitalis*
53080, 53427, 130480, 130692, 130744, 130854	Half mile north of Point Buchon, CA	35.3	-120.9	6	*L. limatula, L. pelta, L. gigantea, L. asmi, A. mitra, L. scabra*
53039, 53135, 53405, 130303	Laguna Bay, CA	35.3	-120.7	4	*L. limatula, L. digitalis, L. pelta, L. gigantea*
53085, 53484, 130627	Cayucos, CA	35.4	-120.9	3	*L. limatula, L. insessa, L. digitalis*
130654	Morro Beach, CA	35.4	-120.9	1	*L. asmi*
53070, 53074, 53180, 130104, 130843	Morro Rock, CA	35.4	-120.9	3	*L. limatula, L. digitalis, L. scabra*
130366	North of Pier, Cayucos Beach, CA	35.4	-120.9	1	*L. scutum*
53143	One mile north of Morro Bay, CA	35.4	-120.9	1	*L. digitalis*
53161, 130860	Morro Bay, CA	35.4	-120.8	2	*L. digitalis, L. scabra*
157406, 157408	Hearst Park, CA	35.6	-121.2	2	*L. pelta, L. limatula*
53236, 53446, 130578, 130855	One mile north of San Simeon, CA	35.6	-121.2	4	*L. pelta, L. insessa, L. digitalis, L. scabra*
130671	One mile south of San Simeon, CA	35.6	-121.2	1	*L. asmi*
53016, 53150, 53388, 130145, 130151, 130156, 130166, 130168, 130175, 130176, 130177, 130325	San Simeon Point, CA	35.6	-121.2	5	*L. limatula, L. digitalis, L. pelta, L. persona, L. scutum*
53013, 53108, 53391, 130680, 130682, 130762	San Simeon, CA	35.6	-121.2	4	*L. limatula, L. pelta, L. asmi, A. mitra*
130228, 130522	Two miles south of San Simeon, CA	35.6	-121.2	2	*L. fenestrata, L. scabra*
130678	Cambria, CA	35.6	-121.1	1	*L. asmi*
53031, 53221, 130241	Moonstone Beach, Cambria, CA	35.6	-121.1	3	*L. limatula, L. pelta, L. fenestrata*
157394, 157395	Five miles north of San Simeon, CA	35.7	-121.3	2	*L. limatula, L. asmi*
53241, 53496	Piedras Blancas Point, CA	35.7	-121.3	2	*L. pelta, L. insessa*
53210	Piedras Blancas, CA	35.7	-121.3	1	*L. instabilis*
157419, 157421, 157423, 157424	National Forest Beach, Cape San Martin, CA	35.9	-121.5	4	*L. limatula, L. scabra, L. pelta, L. persona*
157427	Foot of Jolon Road, CA	36	-121.5	1	*L. limatula*
53086, 53237, 130477, 130637	Soberanes Point, CA	36.4	-121.9	4	*L. limatula, L. pelta, L. gigantea, L. digitalis*
130872	Carmel Bay, CA	36.5	-122	1	*L. scabra*
53532	Granite Creek Canyon off Carmel, CA	36.5	-122	1	*L. instabilis*
53177	Point Lobos, CA	36.5	-122	1	*L. digitalis*
53090	Whaling Station, Point Lobos Reserve, Monterey, CA	36.5	-122	1	*L. limatula*
53521, 130115	Carmel Cove, CA	36.5	-121.9	2	*L. instabilis, L. insessa*
53195	Carmel Point, CA	36.5	-121.9	1	*L. digitalis*
53084, 53181, 130817	Yankee Point, Soberones, CA	36.5	-121.9	3	*L. limatula, L. digitalis, L. scabra*
53408, 53463, 53537, 130537	Asilomar, CA	36.6	-121.9	4	*L. pelta, L. insessa, L. paradigitalis, L. scabra*
130645, 130839	Mission Point, CA	36.6	-121.9	2	*L. digitalis, L. scabra*
53071, 53072, 53073, 53158, 53160, 53185, 53197, 53199, 53226, 53230, 53242, 53324, 53325, 53328, 53377, 53430, 53441, 53471, 53475, 53504, 53511, 53656, 130100, 130112, 130116, 130118, 130138, 130178, 130198, 130288, 130341, 130365, 130370, 130438, 130474, 130475, 130483, 130504, 130505, 130506, 130509, 130511, 130529, 130660, 130662, 130693, 130714, 130734, 130735, 130768, 130783	Monterey, CA	36.6	-121.9	15	*L. limatula, L. digitalis, L. pelta, L. insessa, L. instabilis, L. paradigitalis, T. rosacea, L. depicta, L. triangularis, L. scabra, L. fenestrata, L. scutum, L. gigantea, L. asmi, A. mitra*
130140, 130141, 130157, 130183, 130184, 130362, 130488, 130567	Pacific Grove, CA	36.6	-121.9	7	*L. scutum, L. pelta, L. insessa, L. scabra, L. digitalis, L. gigantea, L. instabilis*
53010, 53172, 53426, 53464, 130489, 130705, 130759, 130833	Point Pinos, Pacific Grove, CA	36.6	-121.9	7	*L. limatula, L. digitalis, L. pelta, L. insessa, L. gigantea, A. mitra, L. scabra*
130628	Spanish Bay, 17 Mile Drive, Monterey, CA	36.6	-121.9	1	*L. digitalis*
130752	Two miles west of Point Pinos, CA	36.6	-121.9	1	*A. mitra*
53247, 130185, 130595	Moss Landing, CA	36.8	-121.8	2	*L. pelta, L. digitalis*
53187, 130117	Davenport, CA	37	-122.2	2	*L. digitalis, L. asmi*
53243, 130234	Near mouth of Scott Creek, Davenport, CA	37	-122.2	2	*L. pelta, L. fenestrata*
130649	Scott Creek, Davenport, CA	37	-122.2	1	*L. digitalis*
53008, 53041, 53079, 53200, 53244, 53416, 53470, 130113, 130132, 130440, 130546, 130579, 130597, 130681, 130697, 130840, 130875	Santa Cruz, CA	37	-122	9	*L. limatula, L. instabilis, L. pelta, L. insessa, L. persona, L. reticulata, L. digitalis, L. asmi, L. scabra*
53376, 130508, 130618	One mile north of Pigeon Point, CA	37.2	-122.4	3	*L. pelta, L. scabra, L. instabilis*
53040, 53211, 53417, 53534, 130205, 130214, 130443, 130492, 130498, 130501, 130548, 130598, 130666, 130701, 130732, 130828	Pigeon Point, CA	37.2	-122.4	10	*L. limatula, L. instabilis, L. pelta, L. fenestrata, L. persona, L. gigantea, L. digitalis, L. asmi, A. mitra, L. scabra*
130459, 130812	Pescadero, CA	37.3	-122.4	2	*L. gigantea, L. scabra*
130207, 130432	Purissima, CA	37.4	-122.4	2	*L. fenestrata, L. persona*
53414	Frenchman's Reef, CA	37.5	-122.5	1	*L. pelta*
53100, 53189, 53194, 53202, 53212, 53323, 53327, 53440, 53449, 130246, 130323, 130368, 130369, 130490, 130528, 130552, 130580, 130604, 130658, 130691, 130726, 130754, 130779, 130813, 130819, 130826	Moss Beach, Half Moon Bay, CA	37.5	-122.5	13	*L. limatula, L. digitalis, L. instabilis, L. pelta, L. spp, L. paradigitalis, L. insessa, L. fenestrata, L. scutum, L. gigantea, L. scabra, L. asmi, A. mitra*
53512, 130777	Pillar Point, Half Moon Bay, CA	37.5	-122.5	2	*L. asmi, A. mitra*
53069, 53129, 53373, 130484, 130702, 130755, 130841	Half Moon Bay, CA	37.5	-122.4	6	*L. limatula, L. digitalis, L. pelta, L. gigantea, A. mitra, L. scabra*
53438	Farallon Islands, CA	37.7	-123	1	*L. pelta*
130120	Southeast Farallon Island, CA	37.7	-123	1	*L. pelta*
130209, 130408	Hunters Point, CA	37.7	-122.4	2	*L. fenestrata, L. persona*
130435	South of Mission, San Francisco, CA	37.7	-122.4	1	*L. persona*
53183	San Francisco Bay, CA	37.7	-122.3	1	*L. digitalis*
53024, 53382, 130414	Fort Point, CA	37.8	-122.5	3	*L. limatula, L. pelta, L. persona*
53140, 53173, 53225, 53393, 53433, 53652, 130180, 130181, 130210, 130212, 130261, 130348, 130424, 130606	Golden Gate, CA	37.8	-122.5	5	*L. digitalis, L. pelta, L. scutum, L. fenestrata, L. paradigitalis*
53332	Rock Point, Point Bonita, CA	37.8	-122.5	1	*L. pelta*
53027, 53118, 53432, 130208, 130340, 130434, 130515, 130516, 130784	San Francisco, CA	37.8	-122.4	7	*L. limatula, L. digitalis, L. pelta, L. fenestrata, L. scutum, L. persona, L. scabra*
53372, 53419, 53429, 53434, 53450, 53469, 130133, 130136, 130142, 130143, 130154, 130169, 130206, 130249, 130339, 130448, 130590, 130679, 130700, 130837	Bolinas Bay, CA	37.9	-122.7	9	*L. pelta, L. insessa, L. asmi, L. scutum, L. paradigitalis, L. fenestrata, L. persona, A. mitra, L. scabra*
130225, 130358, 130359, 130426, 130427, 130428, 130608, 130609, 130610, 130661, 130706	Bolinas, CA	37.9	-122.7	9	*L. fenestrata, L. scutum, L. pelta, L. spp, L. paradigitalis, L. persona, L. asmi, L. digitalis, A. mitra*
130218	Duxbury Point, CA	37.9	-122.7	1	*L. fenestrata*
53122, 130247, 130690, 130834	Duxbury Reef, CA	37.9	-122.7	4	*L. digitalis, L. fenestrata, L. asmi, L. scabra*
130250, 130632, 130703	Stinson Beach, CA	37.9	-122.6	3	*L. fenestrata, L. digitalis, A. mitra*
53009	San Quentin Bay, CA	37.9	-122.5	1	*L. limatula*
130263, 130410, 130513, 130551	Tiburon, CA	37.9	-122.5	3	*L. persona, L. scabra, L. digitalis*
53171, 130229, 130399	Richmond Point, CA	37.9	-122.4	3	*L. digitalis, L. fenestrata, L. persona*
53397, 130231, 130275, 130648, 130844	Drakes Estero, CA	38	-122.9	5	*L. pelta, L. fenestrata, L. persona, L. digitalis, L. scabra*
53240, 53659, 130279, 130499, 130775, 130823	Arch Rock, CA	38	-122.8	6	*L. pelta, L. paradigitalis, L. persona, L. gigantea, A. mitra, L. scabra*
53099, 53451, 130503, 130643	Tomales Point, CA	38.2	-123	4	*L. limatula, L. insessa, L. gigantea, L. digitalis*
53026, 53048, 53081, 53175, 130131, 130163, 130171, 130179, 130431	Tomales Bay, CA	38.2	-122.9	4	*L. limatula, L. digitalis, L. scutum, L. persona*
53246, 130217, 130375, 130629, 130746	Bodega Head, CA	38.3	-123.1	5	*L. pelta, L. fenestrata, L. persona, L. digitalis, A. mitra*
157685, 157686	Half mile south of Mussel Point, CA	38.3	-123.1	2	*L. scabra, L. pelta*
53001, 53370, 130603, 130665, 130729	Horseshoe Cove, CA	38.3	-123.1	5	*L. limatula, L. pelta, L. digitalis, L. asmi, A. mitra*
53224, 53233, 53371, 130388, 130512, 130532, 130533, 130534, 130576, 130624, 130631, 157697, 157698	Mussel Point, CA	38.3	-123.1	6	*L. pelta, L. persona, L. scabra, L. gigantea, L. digitalis, L. asmi*
157688	Bodega Bay, CA	38.3	-123	1	*L. digitalis*
130376, 130535	Bodega Harbor, CA	38.3	-123	2	*L. persona, L. scabra*
53256, 53448, 130230, 130396, 130400, 130573, 130687	Dillon Beach, CA	38.3	-123	6	*L. pelta, L. insessa, L. fenestrata, L. persona, L. digitalis, L. asmi*
53407, 53452, 130226, 130390, 130569, 130820	Carmet, CA	38.4	-123.1	6	*L. pelta, L. insessa, L. fenestrata, L. persona, L. digitalis, L. scabra*
53409, 130216, 130650	Duncans Point, CA	38.4	-123.1	3	*L. pelta, L. fenestrata, L. digitalis*
53257	Goat Rock, CA	38.4	-123.1	1	*L. pelta*
53223, 53383, 53524, 53555, 53655, 130215, 130271, 130367, 130378, 130646, 130733, 130765, 130815	Shell Beach, Bodega Head, CA	38.4	-123.1	9	*L. pelta, L. instabilis, L. fenestrata, L. paradigitalis, L. persona, L. scutum, L. digitalis, A. mitra, L. scabra*
53424, 53553	Shell Beach, Sonoma, CA	38.4	-123.1	2	*L. pelta, A. strigatella*
53508	Stillwater Cove, CA	38.5	-123.3	1	*L. instabilis*
53665, 130523, 130524, 130568	Timber Cove, CA	38.5	-123.3	4	*L. paradigitalis, L. scabra, L. gigantea, L. digitalis*
130469, 130521, 130617, 130659	Horseshoe Point, CA	38.6	-123.4	4	*L. gigantea, L. scabra, L. digitalis, L. asmi*
53531, 130219, 130386, 130652, 130713, 130846	Salt Point, CA	38.6	-123.3	6	*L. instabilis, L. fenestrata, L. persona, L. digitalis, A. mitra, L. scabra*
53186, 53445, 53506, 130220, 130385, 130747, 130838	Del Mar Point, CA	38.7	-123.5	7	*L. digitalis, L. insessa, L. instabilis, L. fenestrata, L. persona, A. mitra, L. scabra*
53193, 53239, 130238, 130740, 130850	Del Mar Ranch, CA	38.7	-123.5	5	*L. digitalis, L. pelta, L. fenestrata, A. mitra, L. scabra*
53149, 53170, 53322, 53437, 130835	Stewart's Point, CA	38.7	-123.4	3	*L. digitalis, L. pelta, L. scabra*
53516, 130202, 130266, 130668	Havens Neck, CA	38.8	-123.6	4	*L. instabilis, L. fenestrata, L. persona, L. asmi*
53510	Gualala, CA	38.8	-123.5	1	*L. instabilis*
53124, 53431, 53505, 130201, 130672, 130763, 130827	Arena Cove, CA	38.9	-123.7	7	*L. digitalis, L. pelta, L. instabilis, L. fenestrata, L. asmi, A. mitra, L. scabra*
53130, 53406, 130264, 130371, 130676	Point Arena, CA	38.9	-123.7	4	*L. digitalis, L. pelta, L. persona, L. asmi*
130593, 130836	Seal Beach rocks near Lighthouse Point, CA	38.9	-123.7	2	*L. digitalis, L. scabra*
53436, 130651, 130862	North of Lighthouse Point, Arena, CA	39	-123.7	3	*L. pelta, L. digitalis, L. scabra*
53461	Near Elk Creek, CA	39.1	-123.7	1	*L. insessa*
53133, 130278, 130664	Near mouth of Elk Creek, South of Elk Creek, CA	39.1	-123.7	3	*L. digitalis, L. persona, L. asmi*
130334	Little River, CA	39.3	-123.8	1	*L. scutum*
130204, 130222, 130239	Mendocino, CA	39.3	-123.8	1	*L. fenestrata*
53156, 53205, 53278, 53515, 130235, 130268, 130412, 130599, 130611, 130625, 130742, 130771, 130856	Russian Gulch, CA	39.3	-123.8	8	*L. digitalis, T. rosacea, L. pelta, L. instabilis, L. fenestrata, L. persona, A. mitra, L. scabra*
53134, 53390, 130236, 130389, 130510	Fort Bragg Landing Beach, CA	39.4	-123.8	5	*L. digitalis, L. pelta, L. fenestrata, L. persona, L. scabra*
53136, 53213, 130153, 130213, 130331, 130382, 130413, 130780	Fort Bragg, CA	39.4	-123.8	7	*L. digitalis, L. instabilis, L. pelta, L. fenestrata, L. scutum, L. persona, A. mitra*
53191	Near Hare Creek, south of Fort Bragg, CA	39.4	-123.8	1	*L. digitalis*
130673	Cape Vizcaino, CA	39.7	-123.8	1	*L. asmi*
53112, 53399, 130232, 130274, 130756, 130864	One mile south of Hardy, CA	39.7	-123.8	6	*L. digitalis, L. pelta, L. fenestrata, L. persona, A. mitra, L. scabra*
130709	1000 yards south of Cape Mendocino, CA	40.4	-124.4	1	*A. mitra*
53218, 130352	Cape Mendocino, CA	40.4	-124.4	2	*L. pelta, L. scutum*
53176, 53467, 130257, 130383, 130656, 130766, 130816	Two miles south of Cape Mendocino, CA	40.4	-124.4	7	*L. digitalis, L. instabilis, L. fenestrata, L. persona, L. asmi, A. mitra, L. scabra*
53167, 130429	Humboldt County, CA	40.7	-124.2	2	*L. digitalis, L. persona*
53317	North Jelly Mid Tidal Zone, Humboldt Bay, CA	40.7	-124.2	1	*L. pelta*
130536	North Jetty, Humboldt Bay, CA	40.7	-124.2	1	*L. scabra*
130227, 130272, 130583	North of Redwood Creek, Orick, CA	41.3	-124.1	3	*L. fenestrata, L. persona, L. digitalis*
130403	North end of Klamath Cove, Requia, CA	41.5	-124.1	1	*L. persona*
130601	North end of False Klamath Cove, Requia, CA	41.6	-124.1	1	*L. digitalis*
130409, 130638	South end of False Klamath Cove, Requia, CA	41.6	-124.1	2	*L. persona, L. digitalis*
53313, 53522, 53527	Crescent City, CA	41.8	-124.2	2	*L. pelta, L. instabilis*
53249, 130245, 130401, 130571	Near CA-OR border, Smith River, CA	41.9	-124.2	3	*L. pelta, L. fenestrata, L. digitalis*
53125, 53425, 130265	Port Orford, OR	42.7	-124.5	3	*L. digitalis, L. pelta, L. persona*
53329	Bridgeport, OR	43	-124	1	*L. pelta*
53184, 53330, 130328, 130433	Coos Bay, OR	43.4	-124.2	4	*L. digitalis, L. pelta, L. scutum, L. persona*
130404	Tillamook, OR	45.5	-123.8	1	*L. persona*
130381	Point Chehalis, WA	46.9	-124.1	1	*L. persona*
53344, 53352	Olympia, WA	47	-122.9	1	*L. pelta*
53420	Tacoma, WA	47.3	-122.4	1	*L. pelta*
130417	Hood Canal, WA	47.6	-123	1	*L. persona*
130262	Rocky Point Dye Inlet, Bremerton, WA	47.6	-122.7	1	*L. persona*
53354, 53401	Waterman Point, WA	47.6	-122.6	1	*L. pelta*
53351	Near Orchard Point, WA	47.6	-122.5	1	*L. pelta*
53139, 130345, 130592	Restoration Point, Bainbridge Island, WA	47.6	-122.5	2	*L. digitalis, L. scutum*
53154, 53339, 130137, 130150, 130221, 130416	Seattle, WA	47.6	-122.3	5	*L. digitalis, L. pelta, L. scutum, L. fenestrata, L. persona*
53028, 53121, 53253, 53338, 53384, 130270, 130439, 130699	Puget Sound, WA	47.8	-122.4	6	*L. limatula, L. digitalis, L. pelta, L. scutum, L. persona, A. mitra*
53519, 130224, 130730	Freshwater Bay, WA	48.1	-123.6	3	*L. instabilis, L. fenestrata, A. mitra*
53164, 53214, 53334, 53347, 53349, 53355	Port Angeles, WA	48.1	-123.4	3	*L. digitalis, L. instabilis, L. pelta*
53120, 53203, 130360, 130361, 130420, 130707	Port Townsend, WA	48.1	-122.8	5	*L. digitalis, L. instabilis, L. scutum, L. persona, A. mitra*
53509, 130394, 130720	Pillar Point near Pysht, WA	48.2	-124.1	3	*L. instabilis, L. persona, A. mitra*
53336	Pillar Point, WA	48.2	-124.1	1	*L. pelta*
53116, 53501, 53520, 130199, 130330, 130392, 130739	Twin Rivers, WA	48.2	-124	6	*L. digitalis, L. instabilis, L. fenestrata, L. scutum, L. persona, A. mitra*
53462	Crescent Bay, WA	48.2	-123.7	1	*L. insessa*
53333, 53514, 130139, 130337, 130354, 130415, 130563, 130719	Port Crescent, WA	48.2	-123.7	6	*L. pelta, L. instabilis, L. scutum, L. persona, L. digitalis, A. mitra*
53507, 53517, 53526, 130721, 130873	Clallam Bay, WA	48.3	-124.3	3	*L. instabilis, A. mitra, L. scabra*
53174, 130704	Straits of Fuca, Vancouver Island, CA	48.3	-124	2	*L. digitalis, A. mitra*
53188, 53196, 53209, 53220, 53552, 130127, 130237	Tatoosh Island, WA	48.4	-124.7	6	*L. digitalis, L. instabilis, L. pelta, A. strigatella, T. testudinalis, L. fenestrata*
53290, 53346, 53350, 53502, 53513, 53518, 53523, 130152, 130273, 130351, 130397, 130425, 130566, 130596	Neah Bay, WA	48.4	-124.6	5	*L. pelta, L. instabilis, L. scutum, L. persona, L. digitalis*
130355	Waddah Island, Neah Bay, WA	48.4	-124.6	1	*L. scutum*
130405	Between Muir and Kirby Creek, Vancouver Island, BC	48.4	-123.9	1	*L. persona*
130335, 130565, 130722	Kirby Creek, Vancouver Island, BC	48.4	-123.9	3	*L. scutum, L. digitalis, A. mitra*
53301, 130344, 130588, 130822	Victoria, BC	48.4	-123.4	3	*L. pelta, L. scutum, L. digitalis*
53337	Oak Bay, WA	48.4	-123.3	1	*L. pelta*
53353	Agate Beach, WA	48.4	-122.9	1	*L. pelta*
53385, 130616	Rosario Beach, WA	48.4	-122.7	2	*L. pelta, L. digitalis*
130373	False Bay, San Juan Island, WA	48.5	-123.1	1	*L. persona*
53201, 53342, 53343, 130135, 130147, 130149, 130277, 130430, 130639	San Juan Island, WA	48.5	-123.1	5	*L. instabilis, L. pelta, L. scutum, L. persona, L. digitalis*
130374, 130384, 130584, 130619, 130745	Brown Island, Friday Harbor, WA	48.5	-123	3	*L. persona, L. digitalis, A. mitra*
130710, 130718	Friday Harbor, WA	48.5	-123	1	*A. mitra*
53400, 130636	Minnesota Reef, San Juan Island, WA	48.5	-123	2	*L. pelta, L. digitalis*
53413	North side of Brown Island, WA	48.5	-123	1	*L. pelta*
53340, 130602	Davis Bay, Lopez Island, WA	48.5	-122.9	2	*L. pelta, L. digitalis*
53168, 53348, 130343, 130372	Anacortes, WA	48.5	-122.6	4	*L. digitalis, L. pelta, L. scutum, L. persona*
53341, 130770	Garrison Bay, San Juan Island, WA	48.6	-123.2	2	*L. pelta, A. mitra*
53003	San Hippolita Point, Baja	48.6	-123.2	1	*L. limatula*
130574	Clo-oose, Vancouver Island, BC	48.7	-124.8	1	*L. digitalis*
130280	Waldron Island, WA	48.7	-123	1	*L. persona*
53291, 130582, 130874	Crescent Beach, WA	48.7	-122.9	3	*L. pelta, L. digitalis, L. scabra*
130441	Sucia Islands, WA	48.8	-122.9	1	*L. persona*
53404, 53412, 53428	Vancouver, BC	49.3	-123.1	1	*L. pelta*
130223, 130406	West Vancouver Island, BC	49.5	-126.6	2	*L. fenestrata, L. persona*
130418	Vancouver Island, BC	49.7	-125.8	1	*L. persona*
53126, 53320, 130200, 130342, 130407	Beaver Cove, Vancouver Island, BC	50.5	-126.9	5	*L. digitalis, L. pelta, L. fenestrata, L. scutum, L. persona*
53162	Chatham Sound, Queen Charlotte Island, BC	53.3	-132.1	1	*L. digitalis*
130349, 130736	East coast of Graham Island, BC	53.4	-131.9	2	*L. scutum, A. mitra*
130737	False Narrows, Unimak Island, AK	54.8	-163.4	1	*A. mitra*
130356, 130357, 130398	Annette Island, AK	55.1	-131.4	2	*L. scutum, L. persona*
130363	Popof Island, AK	55.3	-160.4	1	*L. scutum*
130336, 130391	Black Point, CA	38.7	-123.4	2	*L. scutum, L. persona*
130377	Pavalof Bay, Chichagof Island, AK	55.5	-161.5	1	*L. persona*
53292, 130203, 130267, 130716	Red Bluff Bay, Baranoff Island, AK	56.9	-134.8	4	*L. pelta, L. fenestrata, L. persona, A. mitra*
130333, 130712	Sitka, AK	57.1	-135.3	2	*L. scutum, A. mitra*
53068, 130121, 130444, 130715	Bear Bay, Baranoff Island, AK	57.5	-135.5	4	*L. limatula, A. funiculata, L. persona, A. mitra*
53208, 53319, 130724	Chatham, AK	57.5	-134.9	3	*L. instabilis, L. pelta, A. mitra*
130324	Mole Harbor, Admiralty Island, AK	57.7	-134.1	1	*L. scutum*
130727, 130741	Kodiak, AK	57.8	-152.4	1	*A. mitra*
53620	Paralof Bay, AK	57.9	-135.8	1	*L. paradigitalis*
53293, 53306, 53316, 130329	Windfall Harbor, Admiralty Island, AK	57.9	-134.3	2	*L. pelta, L. scutum*
130353	Head of Port Frederick, AK	58.1	-135.6	1	*L. scutum*
53151, 53304	Hoonah, Chichagoff Island, AK	58.1	-135.4	2	*L. digitalis, L. pelta*
130347	Hawk Inlet, AK	58.1	-134.8	1	*L. scutum*
130276	Green Cove, Admiralty Island, AK	58.2	-134.3	1	*L. persona*
130364	Point Louisa, AK	58.4	-134.7	1	*L. scutum*
53310, 130346, 130445	Glacier Bay, AK	58.8	-136.3	3	*L. pelta, L. scutum, L. persona*
53309, 130387, 130393	Yakutat, AK	59.5	-139.7	2	*L. pelta, L. persona*
53380	Cape Yakataga, AK	60.1	-142.4	1	*L. pelta*
53138, 53294, 53394	AK	NA	NA	3	*L. digitalis, C. concentrica, L. pelta*
53002, 53107	Baja	NA	NA	1	*L. limatula*
53299	British Columbia	NA	NA	1	*L. pelta*
53435, 130402, 130550, 130663	CA	NA	NA	4	*L. pelta, L. persona, L. digitalis, L. asmi*
53486	Half Moon Bay OR Santa Barbara, CA	NA	NA	1	*L. insessa*
53055	Halfway Reef Cliffs, Baja	NA	NA	1	*L. limatula*
53047, 53141, 53422, 130332, 130350, 130423, 130622	Lagoon Head, Baja	NA	NA	5	*L. limatula, L. digitalis, L. pelta, L. scutum, L. persona*
53033	Mexico	NA	NA	1	*L. limatula*
53540	Moreno, CA	NA	NA	1	*L. depicta*
53528	Northern CA	NA	NA	1	*L. instabilis*
53503, 130575	OR	NA	NA	2	*L. instabilis, L. digitalis*
53295	Southwest AK	NA	NA	1	*L. pelta*
53466	Trinidad to Magdalena Bay, Monetery, CA	NA	NA	1	*L. insessa*
53335, 53530	WA	NA	NA	2	*L. pelta, L. digitalis*
A list and count of the species present at each unique locality. Rounded coordinates associated with each site are provided.					

**Table 3:** Species range minima/maxima and number of individuals per species

**species**	**max latitudinal range**	**min latitudinal range**	**number of individuals**	**UCMP specimen numbers**
*Lottia limatula*	57.5	27.7	1626	53000, 53001, 53052, 53003, 53005, 53006, 53007, 53008, 53009, 53010, 53011, 53013, 53016, 53017, 53018, 53019, 53020, 53021, 53022, 53023, 53024, 53025, 53026, 53027, 53028, 53029, 53030, 53031, 53033, 53034, 53036, 53038, 53039, 53040, 53041, 53042, 53043, 53044, 53045, 53046, 53047, 53048, 53049, 53050, 53051, 53053, 53054, 53055, 53056, 53057, 53058, 53059, 53060, 53061, 53062, 53063, 53064, 53065, 53066, 53067, 53068, 53069, 53070, 53071, 53072, 53073, 53074, 53075, 53077, 53078, 53079, 53080, 53081, 53083, 53084, 53085, 53086, 53087, 53088, 53089, 53090, 53091, 53092, 53093, 53094, 53095, 53097, 53099, 53100, 53101, 53102, 53105, 53107, 53108, 53109, 53110, 53668, 53676, 130038, 130100, 130101, 130102, 130103, 130104, 130105, 130155, 130171, 130173, 130176, 130179, 130540, 130541, 130876, 157394, 157408, 157411, 157419, 157427, 157470, 53002, 53037
*Lottia digitalis*	58.1	26	2276	53111, 53112, 53113, 53114, 53115, 53116, 53117, 53118, 53119, 53120, 53121, 53122, 53123, 53124, 53125, 53126, 53128, 53129, 53130, 53131, 53132, 53133, 53134, 53135, 53136, 53137, 53138, 53139, 53140, 53141, 53142, 53143, 53144, 53145, 53146, 53147, 53148, 53149, 53150, 53151, 53152, 53153, 53154, 53155, 53156, 53158, 53159, 53160, 53161, 53162, 53163, 53164, 53165, 53166, 53167, 53168, 53169, 53170, 53171, 53172, 53173, 53174, 53175, 53176, 53177, 53178, 53179, 53180, 53181, 53182, 53183, 53184, 53185, 53186, 53187, 53188, 53189, 53191, 53192, 53193, 53194, 53195, 53196, 53197, 53530, 53634, 53654, 53670, 53671, 53672, 53678, 53679, 53680, 130163, 130166, 130170, 130172, 130181, 130184, 130185, 130186, 130519, 130534, 130544, 130547, 130548, 130549, 130550, 130551, 130552, 130553, 130554, 130555, 130556, 130558, 130559, 130560, 130561, 130562, 130563, 130564, 130565, 130566, 130568, 130569, 130570, 130571, 130572, 130573, 130574, 130575, 130576, 130577, 130578, 130579, 130580, 130581, 130582, 130583, 130584, 130586, 130588, 130589, 130591, 130592, 130593, 130595, 130596, 130597, 130598, 130599, 130600, 130601, 130602, 130603, 130604, 130605, 130606, 130607, 130609, 130611, 130612, 130613, 130614, 130615, 130616, 130617, 130619, 130620, 130621, 130622, 130623, 130624, 130625, 130626, 130627, 130628, 130629, 130630, 130631, 130632, 130634, 130635, 130636, 130637, 130638, 130639, 130640, 130641, 130643, 130645, 130646, 130647, 130648, 130649, 130650, 130651, 130652, 130653, 130822, 157688
*Lottia pelta*	60.1	31.6	2047	53190, 53199, 53217, 53218, 53219, 53220, 53221, 53222, 53223, 53224, 53225, 53226, 53227, 53228, 53229, 53230, 53231, 53232, 53233, 53234, 53236, 53237, 53238, 53239, 53240, 53241, 53242, 53243, 53244, 53245, 53246, 53247, 53249, 53250, 53253, 53254, 53256, 53257, 53258, 53260, 53262, 53278, 53280, 53290, 53291, 53292, 53293, 53295, 53299, 53301, 53303, 53304, 53306, 53309, 53310, 53311, 53312, 53313, 53315, 53316, 53317, 53319, 53320, 53321, 53322, 53323, 53324, 53325, 53326, 53327, 53328, 53329, 53330, 53331, 53332, 53333, 53334, 53335, 53336, 53337, 53338, 53339, 53340, 53341, 53343, 53344, 53346, 53347, 53348, 53349, 53350, 53351, 53352, 53353, 53354, 53355, 53364, 53370, 53371, 53372, 53373, 53374, 53375, 53376, 53377, 53378, 53379, 53380, 53381, 53382, 53383, 53384, 53385, 53386, 53387, 53388, 53389, 53390, 53391, 53393, 53394, 53395, 53396, 53397, 53398, 53399, 53400, 53401, 53404, 53405, 53406, 53407, 53408, 53409, 53411, 53412, 53413, 53414, 53415, 53416, 53417, 53418, 53419, 53420, 53421, 53422, 53423, 53424, 53425, 53426, 53427, 53428, 53429, 53430, 53431, 53432, 53433, 53434, 53435, 53436, 53437, 53438, 53439, 53440, 53441, 53681, 53682, 130120, 130128, 130129, 130132, 130141, 130142, 130143, 130145, 130146, 130148, 130151, 130153, 130168, 130178, 130180, 130382, 130401, 130426, 157406, 157423, 157460, 157490, 157686
*Lottia instabilis*	57.5	34.4	204	53200, 53201, 53202, 53203, 53208, 53209, 53210, 53211, 53212, 53213, 53214, 53342, 53467, 53501, 53502, 53503, 53504, 53505, 53506, 53507, 53508, 53509, 53510, 53511, 53513, 53514, 53515, 53516, 53517, 53518, 53519, 53520, 53521, 53522, 53523, 53524, 53525, 53526, 53527, 53528, 53531, 53532, 53534, 130567, 130618
*Testudinalia testudinalis*	48.4	27.7	10	53204, 130127
*Tectura rosacea*	39.3	32.4	48	53205, 53206, 53207, 53215, 53216, 130112, 130119, 130124, 130282, 130284, 130285, 130286, 130287, 130288, 130783
*Cryptobranchia concentrica*	NA	NA	1	53294
*Acmaea strigatella*	48.4	33.2	21	53302, 53552, 53553
*Lottia spp*	37.9	37.5	2	53327, 130426
*Lottia paradigitalis*	57.9	26	283	53327, 53537, 53551, 53620, 53637, 53639, 53641, 53655, 53656, 53659, 53665, 53669, 130169, 130187, 130188, 130189, 130192, 130194, 130195, 130255, 130424, 130426, 130545, 130557, 130585, 130590, 130610
*Lottia insessa*	48.2	31.6	771	53369, 53443, 53444, 53445, 53446, 53448, 53449, 53450, 53451, 53452, 53461, 53462, 53463, 53464, 53465, 53466, 53468, 53469, 53470, 53471, 53472, 53473, 53474, 53475, 53476, 53478, 53479, 53480, 53481, 53482, 53483, 53484, 53485, 53486, 53487, 53488, 53489, 53490, 53491, 53492, 53493, 53494, 53495, 53496, 53497, 53498, 53499, 53628, 53633, 130113, 130115, 130157, 157467
*Lottia asmi*	40.4	30.6	262	53512, 130117, 130133, 130608, 130654, 130656, 130658, 130659, 130660, 130661, 130662, 130663, 130664, 130665, 130666, 130667, 130668, 130669, 130670, 130671, 130672, 130673, 130675, 130676, 130677, 130678, 130679, 130680, 130681, 130682, 130683, 130684, 130685, 130686, 130687, 130688, 130689, 130690, 130691, 130692, 130693, 130694, 130695, 130696, 130697, 130698, 157395, 157698, 131502
*Lottia depicta*	36.6	31.7	76	53535, 53536, 53540, 53541, 53542, 53543, 53544, 53545, 53546, 53547, 53548, 53549, 53550, 130108, 130116
*Lottia fenestrata*	56.9	26.7	620	53538, 53539, 53554, 53555, 53643, 53644, 53645, 53646, 53647, 53648, 53649, 53650, 53651, 53660, 53667, 130193, 130196, 130198, 130199, 130200, 130201, 130202, 130203, 130204, 130205, 130206, 130207, 130208, 130209, 130210, 130211, 130212, 130213, 130214, 130215, 130216, 130217, 130218, 130219, 130220, 130221, 130222, 130223, 130224, 130225, 130226, 130227, 130228, 130229, 130230, 130231, 130232, 130233, 130234, 130235, 130236, 130237, 130238, 130239, 130240, 130241, 130242, 130243, 130244, 130245, 130246, 130247, 130248, 130249, 130250, 130251, 130252, 130253, 130254, 130256, 130257, 130258, 130259, 130260, 130261, 157380
*Lottia dalliana*	34.5	26	137	53557, 53560, 53564, 53565, 53571, 53572, 53574, 53575, 53583, 53588, 53605, 130106, 130281, 130542, 130543
*Lottia mesoleuca*	31	24.1	89	53562, 53569, 53582, 53586, 53589, 53591, 53593, 53594, 53598, 53611, 53615
*Lottia stanfordiana*	30.4	30.1	7	53577, 53584, 53606
*Lottia concreta*	31	31	12	53578
*Lottia gigantea*	38.6	27.7	226	53625, 130293, 130294, 130296, 130299, 130300, 130301, 130302, 130303, 130304, 130305, 130306, 130307, 130308, 130309, 130310, 130311, 130312, 130313, 130316, 130317, 130318, 130321, 130322, 130438, 130454, 130458, 130459, 130460, 130461, 130463, 130464, 130465, 130466, 130467, 130468, 130469, 130471, 130474, 130475, 130477, 130479, 130480, 130481, 130482, 130483, 130484, 130488, 130489, 130490, 130491, 130492, 130495, 130496, 130498, 130499, 130500, 130501, 130502, 130503, 130506, 130524, 130533
*Lottia scabra*	48.7	26.7	858	53627, 53632, 130109, 130114, 130138, 130174, 130183, 130190, 130504, 130505, 130507, 130508, 130509, 130510, 130511, 130512, 130513, 130514, 130515, 130516, 130517, 130518, 130521, 130522, 130523, 130525, 130526, 130527, 130528, 130529, 130530, 130531, 130532, 130535, 130536, 130537, 130538, 130539, 130587, 130784, 130786, 130794, 130812, 130813, 130814, 130815, 130816, 130817, 130819, 130820, 130823, 130824, 130825, 130826, 130827, 130828, 130829, 130830, 130831, 130832, 130833, 130834, 130835, 130836, 130837, 130838, 130839, 130840, 130841, 130842, 130843, 130844, 130845, 130846, 130848, 130849, 130850, 130851, 130852, 130853, 130854, 130855, 130856, 130857, 130858, 130859, 130860, 130861, 130862, 130863, 130864, 130865, 130866, 130867, 130868, 130869, 130870, 130872, 130873, 130874, 130875, 157410, 157421, 157429, 157685, 157697
*Lottia scutum*	58.8	27.7	761	53638, 53652, 53662, 53675, 130131, 130135, 130136, 130137, 130139, 130140, 130147, 130149, 130150, 130152, 130154, 130175, 130270, 130323, 130324, 130325, 130326, 130327, 130328, 130329, 130330, 130331, 130332, 130333, 130334, 130335, 130336, 130337, 130338, 130339, 130340, 130341, 130342, 130343, 130344, 130345, 130346, 130347, 130348, 130349, 130350, 130351, 130352, 130353, 130354, 130355, 130356, 130357, 130358, 130359, 130360, 130361, 130362, 130363, 130364, 130365, 130366, 130367, 130368, 130369, 130370, 130421, 130427, 130451, 153640
*Lottia persona*	59.5	27.7	1152	53661, 130134, 130156, 130158, 130177, 130262, 130263, 130264, 130265, 130266, 130267, 130268, 130269, 130271, 130272, 130273, 130274, 130275, 130276, 130277, 130278, 130279, 130280, 130371, 130372, 130373, 130374, 130375, 130376, 130377, 130378, 130379, 130381, 130383, 130384, 130385, 130386, 130387, 130388, 130389, 130390, 130391, 130392, 130393, 130394, 130395, 130396, 130397, 130398, 130399, 130400, 130402, 130403, 130404, 130405, 130406, 130407, 130408, 130409, 130410, 130411, 130412, 130413, 130414, 130415, 130416, 130417, 130418, 130420, 130422, 130423, 130425, 130428, 130429, 130430, 130431, 130432, 130433, 130434, 130435, 130439, 130440, 130441, 130442, 130443, 130444, 130445, 130446, 130447, 130448, 130449, 130450, 130452, 157424
*Acmaea funiculata*	57.5	32.4	108	130110, 130111, 130121, 130191
*Lottia triangularis*	36.6	36.6	2	130118
*Lottia discus*	30.3	30.3	3	130122
*Lottia paleacea*	27.7	27.7	9	130123
*Lottia atrata*	22.9	22.9	8	130125
*Acmaea turveri*	31	31	19	130126
*Acmaea mitra*	57.8	31.6	396	130520, 130699, 130700, 130701, 130702, 130703, 130704, 130705, 130706, 130707, 130708, 130709, 130710, 130711, 130712, 130713, 130714, 130715, 130716, 130717, 130718, 130719, 130720, 130721, 130722, 130723, 130724, 130725, 130726, 130727, 130728, 130729, 130730, 130731, 130732, 130733, 130734, 130735, 130736, 130737, 130739, 130740, 130741, 130742, 130744, 130745, 130746, 130747, 130748, 130749, 130750, 130751, 130752, 130753, 130754, 130755, 130756, 130757, 130758, 130759, 130760, 130761, 130762, 130763, 130764, 130765, 130766, 130767, 130768, 130769, 130770, 130771, 130772, 130773, 130774, 130775, 130776, 130777, 130778, 130779, 130780, 130781, 130782
*Lottia (?) reticulata*	37	37	1	130546
A list of the range minima and maxima for the thirty species represented in the dataset. UCMP specimen numbers and the number of individuals in each species are provided.				

**Table 4:** Measurement validation

**UCMP specimen number**	**UCMP locality number**	**object number**	**hand-measured length (mm)**	**hand-measured width (mm)**	**AutoMorph object ID**	**AutoMorph length (major axis; mm, rounded)**	**AutoMorph width (minor axis; mm, rounded)**
53003	2782-	2	15.8	12.7	UCMP.53003_obj00002	15.565	12.133
53003	2782-	3	17.7	14.3	UCMP.53003_obj00003	16.65	14.174
53003	2782-	4	13.3	10.7	UCMP.53003_obj00004	12.787	10.54
53003	2782-	5	12.8	9.9	UCMP.53003_obj00005	12.379	9.484
53003	2782-	6	13.5	10.5	UCMP.53003_obj00006	13.359	10.22
53003	2782-	7	18.5	14.2	UCMP.53003_obj00007	18	13.907
53003	2782-	8	13.3	10.4	UCMP.53003_obj00008	12.986	9.892
53003	2782-	9	18.6	13.6	UCMP.53003_obj00009	18.324	13.948
53003	2782-	10	5.3	3.3	UCMP.53003_obj00010	5	3.72
53003	2782-	11	16.5	13.5	UCMP.53003_obj00011	16.458	12.952
53003	2782-	12	13.6	10.7	UCMP.53003_obj00012	13.468	10.603
53003	2782-	13	16.4	13.1	UCMP.53003_obj00013	15.933	12.531
53003	2782-	14	16.2	12.7	UCMP.53003_obj00014	15.741	11.853
53003	2782-	15	15.6	12.7	UCMP.53003_obj00015	15.282	11.826
53003	2782-	16	14.2	11.5	UCMP.53003_obj00016	13.827	11.266
53003	2782-	17	17	13.5	UCMP.53003_obj00017	16.619	12.881
53003	2782-	18	14.3	11.2	UCMP.53003_obj00018	14.488	11.029
130454	E873	1	88.6	69	UCMP.130454_obj00001	80.905	61.38
130454	E873	2	92.7	78.8	UCMP.130454_obj00002	89.241	73.915
130454	E873	3	57.4	46.5	UCMP.130454_obj00003	54.414	43.269
130454	E873	5	67.1	51.1	UCMP.130454_obj00005	63.625	48.199
130454	E873	6	93.5	74.5	UCMP.130454_obj00006	88.931	70.134
53445	E425	2	10.9	7.8	UCMP.53445_obj00002	10.246	7.392
53445	E425	3	10.3	7	UCMP.53445_obj00003	10.06	6.393
53445	E425	4	16.8	12.5	UCMP.53445_obj00004	16.428	12.216
53445	E425	5	9.6	7.6	UCMP.53445_obj00005	9.415	7.425
53445	E425	6	11.3	8.5	UCMP.53445_obj00006	11.005	8.296
53445	E425	7	9.2	7	UCMP.53445_obj00007	8.246	6.337
53445	E425	8	18.9	12.7	UCMP.53445_obj00008	18.644	12.63
53445	E425	9	12.7	9.1	UCMP.53445_obj00009	12.551	9.08
53445	E425	10	9.2	7.2	UCMP.53445_obj00010	8.998	6.353
53445	E425	11	18.2	12.5	UCMP.53445_obj00011	17.886	12.449
53445	E425	12	11.5	8.8	UCMP.53445_obj00012	11.114	8.553
53445	E425	13	12.3	9	UCMP.53445_obj00013	12.048	8.789
53445	E425	14	19.7	12.6	UCMP.53445_obj00014	19.37	12.45
53445	E425	15	18.8	13.2	UCMP.53445_obj00015	17.913	12.748
53445	E425	16	12	8.9	UCMP.53445_obj00016	11.706	8.629
53445	E425	17	19.2	13.1	UCMP.53445_obj00017	19.006	13.084
53445	E425	18	20.6	13.6	UCMP.53445_obj00018	20.391	13.146
53445	E425	19	11.9	8.1	UCMP.53445_obj00019	11.737	8.01
53445	E425	20	9	6.6	UCMP.53445_obj00020	8.656	6.529
A comparison of hand-measured major axis length and minor axis length with corresponding *AutoMorph* outputs for 41 specimens. Refer to Fig. 4 for a linear regression of the two measurement types.							

**Table 5:** Locality information mismatches

**UCMP specimen number(s)**	**UCMP locality number(s)**	**specimen-associated locality string**	**database-associated locality string**
130273	E6920	Neah Bay, WA	Port Orford, OR
130561	E6853	Cabrillo Beach, CA	Mexico
130562	E6852	Venice CA	Mexico
130614	IP10950	San Nicolas Island, CA	Washington
Three UCMP locality numbers assigned to specimens upon curation have associated metadata that do not match current metadata housed on the UCMP online database. These specimen-associated locality numbers and their metadata are listed, and the metadata contained in the online database is provided for comparison.			
